# Supplementary material for: Resolution of the ordinal phylogeny of mosses using targeted exons from organellar and nuclear genomes
Source: Nat Commun. 2019 Apr 2;10:1485. doi: 10.1038/s41467-019-09454-w (PMC6445109; doi:10.1038/s41467-019-09454-w)
Supplement: Supplementary file 1 — Supplementary Information [file 41467_2019_9454_MOESM1_ESM.pdf]

Supplementary Table 1. Voucher information for the sampled mosses and liverwort outgroups analyzed in this study. Classification followed Goffinet et al. (1) except for *Thamnobryum hispidum* [see Stech et al. (2)]. Samples using transcriptome data from the 1KP project (3) or Devos et al. (4) are marked with "*" and "**", respectively. Samples lacking locality data are derived from the 1KP project (3). Herbarium codes: **CONN**-University of Connecticut; **DUKE**-Duke University; **H**-University of Helsinki; **LG**-Université de Liège; **PE**-Institute of Botany, Chinese Academy of Sciences; **MUR**-Murray State University; **NY**-The New York Botanical Garden.

**Genus**	**Species**	**Family**	**Collection #**	**Locality**	**Herbarium**
Achrophyllum	*magellanicum*	Daltoniaceae	Goffinet 12899	Chile, Antártica Chilena	CONN
Alophosia	*azorica*	Polytrichaceae	Vanderpoorten & Patiño	Portugal, Azorres	LG
Ambuchanania	*leucobryoides* **	Ambuchananiaceae	Tyson 1160	Australia, Tasmania	DUKE
Andreaea	*wilsonii*	Andreaeaceae	Goffinet 12835	Chile, Llanquihue	CONN
Andreaea	*rothii*	Andreaeaceae	Goffinet 10586	USA, Connecticut	CONN
Andreaea	*rupestris* *	Andreaeaceae	Larsson AL604		UPS
Andreaeobryum	*macrosporum*	Andreaeobryaceae	Rupp 22 July 1999	USA, Alaska	CONN
Anomodon	*attenuatus*	Anomodontaceae	Goffinet 10590	USA, Connecticut	CONN
Aphanorrhegma	*serratum*	Funariaceae	Buck 49500	USA, Arkansas	NY
Atrichopsis	*compressa*	Polytrichaceae	Buck 58997	Chile, Antártica Chilena	NY
Atrichum	*angustatum*	Polytrichaceae	Goffinet 10582	USA, Connecticut	CONN
Aulacomnium	*palustre*	Aulacomniaceae	Goffinet 11701	USA, Connecticut	CONN
Aulacomnium	*heterostichum*	Aulacomniaceae	Goffinet 11801	USA, West Virginia	CONN
Baldwiniella	*kealeensis*	Neckeraceae	Shevock 44587	USA, Hawaii	CONN
Bartramia	*pomiformis*	Bartramiaceae	Goffinet 10587	USA, Connecticut	CONN
Bazzania	*trilobata*	Lepidoziaceae	Goffinet 10577	USA, Connecticut	CONN
Bazzania	*trilobata* *	Lepidoziaceae	Stevenson		
Bescherellia	*brevifolia*	Hypnodendraceae	Streiman 46048	Australia, Queensland	NY
Bescherellia	*elegantissima*	Hypnodendraceae	Bell 03.11.08.002	New Caledonia	H
Blindia	*acuta*	Seligeriaceae	Buck 61491	Canada, New brunswick	NY
Braithwaitea	*sulcata*	Braithwaiteaceae	Streimann 522853	Australia, Queensland	NY
Braunia	*secunda*	Hedwigiaceae	Hax 02	Mexico, San Miguel Ajusco	CONN

Bruchia	*vogesiaca*	Bruchiaceae	Goffinet 11124	USA, North Carolina	CONN
Bryoxiphium	*norvegicum*	Bryoxiphiaceae	Hax 31	Mexico, Distrito Federal	CONN
Bryum	*argenteum*	Bryaceae	Patiño s.n.	Spain, Tenerife	MUR
Buxbaumia	*aphylla* 1[*]	Buxbaumiaceae	Larsson AL606		UPS
Buxbaumia	*aphylla* 2	Buxbaumiaceae	Goffinet March 5, 2012	USA, Connecticut	CONN
Calyptothecium	*pinnatum*	Pterobryaceae	Akiyama 23246	China, Taiwan	CONN
Calyptrochaeta	*asplenoides*	Daltoniaceae	Goffinet 11626	South Africa, Western Cape	CONN
Canalohypopterygium	*tamariscinum*	Hypopterygiaceae	Shevock 44068	New Zealand, Nelson	CONN
Catharomnion	*ciliatum*	Hypopterygiaceae	Shevock 44144	New Zealand, Nelson	CONN
Catoscopium	*nigritum*	Catoscopiaceae	Hedenäs September 16, 2013	Sweden, Västmanland	CONN
Ceratodon	*purpureus*	Ditrichaceae	Goffinet August 11, 2014	USA, Connecticut	CONN
Chamaebryum	*pottioides*	Gigaspermaceae	Goffinet et al. 11503	South Africa, Western Cape	CONN
Chamaebryum	*pottioides*	Gigaspermaceae	Goffinet 11517	South Africa, Western Cape	CONN
Cladomnion	*ericoides*	Ptychomniaceae	Shevock 44252	New Zealand, Ruapehu District	CONN
Costesia	*macrocarpa*	Gigaspermaceae	Larrain 36906	Chile, Valparaíso Region	CONN
Cyathophorum	*hookerianum*	Hypopterygiaceae	Akiyama 23051	Japan, Kochi Pref.	CONN
Cyptodontopsis	*leveillei*	Cryphaeaceae	Shevock J. 43329	China, Yunnan	CONN
Cyrtopodendron	*vieillardii*	Pterobryellaceae	B. Shaw 16878	New Caledonia, Southern province	DUKE/CONN
Daltonia	*latimarginata*	Daltoniaceae	Carter 7800	Réunion	CONN
Dendrocyathophorum	*decolyi*	Hypopterygiaceae	Jia Y. 10235	China, Chongqing	PE
Dendrohypopterygium	*filiculiforme*	Hypopterygiaceae	Shevock 43973	New Zealand, Nelson-Marlborough	CONN
Dendroligotrichum	*dendroides*	Polytrichaceae	Goffinet 12693	Chile, Los Lagos	CONN
Dichelodontium	*nitidum*	Ptychomniaceae	Shevock 44391	New Zealand, Nelson-Marlborough	CONN
Dicranum	*scoparium*	Dicranaceae	Goffinet 10584	USA, Connecticut	CONN
Diphyscium	*foliosum* 1	Diphysciaceae	Goffinet May, 2012	USA, Connecticut	CONN
Diphyscium	*foliosum* 2[*]	Diphysciaceae	Rothfels & B. Shaw 4153	USA, North Carolina	DUKE
Discelium	*nudum*	Disceliaceae	Dynesius	Sweden, Västerbotten	CONN

Distichophyllum	*pulchellum*	Daltoniaceae	Shevock 43905	New Zealand, Nelson-Marlborough	CONN
Ditrichum	*pallidum*	Ditrichaceae	J. Shaw 17115	USA, North Carolina	DUKE
Drummondia	*prorepens*	Drummondiaceae	Goffinet 11155	USA, North Carolina	CONN
Encalypta	*intermedia*	Encalyptaceae	Shevock 27671	USA, Nevada	NY
Entosthodon	*obtusus*	Funariaceae	Holyoak 04-87	Ireland, Connacht	CONN
Eosphagnum	*inretortum* **	Ambuchananiaceae	B. Shaw 13746	Chile, Antártica Chilena	DUKE/CONN
Euptychium	*cuspidatum*	Ptychomniaceae	B. Shaw 17262	New Caledonia, Northern province	CONN
Euptychium	*setigerum*	Ptychomniaceae	B. Shaw 17698	Fiji, Namosi	DUKE/CONN
Fissidens	*bushii*	Fissidentaceae	Goffinet 11156	USA, North Carolina	CONN
Fissidens	*dubius*	Fissidentaceae	Goffinet 11725	USA, West Virginia	CONN
Flatbergium	*sericeum* **	Flatbergiaceae	Ho 12-228	Malaysia, Peninsular Malaysia	DUKE
Flatbergium	*novo-caledoniae* **	Flatbergiaceae	B. Shaw 17137	New Caledonia, Northern province	DUKE
Funaria	*hygrometrica*	Funariaceae	Goffinet 9344	USA, North Carolina	CONN
Garovaglia	*elegans*	Ptychomniaceae	Shevock 41443	China, Taiwan	CONN
Gigaspermum	*repens*	Gigaspermaceae	Goffinet 11535	South Africa	CONN
Gigaspermum	*repens*	Gigaspermaceae	Goffinet et al. 11468	South Africa	CONN
Glyphothecium	*sciuroides*	Ptychomniaceae	Dalton s.n.	Australia, Tasmania	CONN
Goniobryum	*subbasilare*	Rhizogoniaceae	Goffinet 11058	Chile, Antártica Chilena	CONN
Grimmia	*pulvinata*	Grimmiaceae	Goffinet 11118	Belgium, Namur	CONN
Hampeella	*alaris*	Ptychomniaceae	Dalton s.n.	Australia, Tasmania	CONN
Hedwigia	*ciliata*	Hedwigiaceae	Goffinet 10583	USA, Connecticut	CONN
Hedwigidium	*integrifolium*	Hedwigiaceae	Goffinet 10169	South Africa, Northern Cape	CONN
Hemiragis	*aurea*	Pilotrichaceae	Goffinet 5139	Puerto Rico, SE of San Juan	CONN
Hookeria	*acutifolia*	Hookeriaceae	J. Shaw 17129	USA, North Carolina	DUKE
Hydropogonella	*gymnostoma*	Sematophyllaceae	Akiyama 22711	Japan (imported)	CONN
Hymenodon	*pilifer*	Orthodontiaceae	Dalton August 2012	Australia, Marriotts Falls	CONN
Hymenodontopsis	*mnioides*	Aulacomniaceae	Goffinet 11044	Chile, Antártica Chilena	CONN

Hypnodendron	*vitiense*	Hypnodendraceae	Dalton Aug. 2012	Australia, Marriotts Falls	CONN
Hypopterygium	*didictyon*	Hypopterygiaceae	Goffinet 11049	Chile, Antártica Chilena	CONN
Jaegerina	*solitaria*	Pterobryaceae	Goffinet 11972	Madagascar, Province Diego Suarez	CONN
Lepidopilum	*scabrisetum*	Pilotrichaceae	Lavocat 15404	Guadeloupe	CONN
Leptobryum	*pyriforme*	Meesiaceae	Goffinet 10183	Chile, Antártica Chilena	CONN
Leptostomum	*menziesii*	Leptostomataceae	Buck 61059	Chile, Antártica Chilena	NY
Leptotheca	*gaudichaudii*	Orthodontiaceae	Buck 60770	Chile, Antártica Chilena	NY
Leucobryum	*albidum*	Leucobryaceae	Goffinet 11681	USA, Connecticut	CONN
Leucolepis	*acanthoneuron*	Mniaceae	Medina 1172	USA, California	CONN
Leucoloma	*rehmanii*	Dicranaceae	Goffinet 10263	South Africa, Western Cape	CONN
Lopidium	*struthiopteris*	Hypopterygiaceae	Goffinet 11616	South Africa, Western Cape	CONN
Lorentziella	*imbricata*	Gigaspermaceae	Rushing April 14, 2013	USA, Texas	CONN
Macromitrium	*sulcatum*	Orthotrichaceae	Goffinet 12447	Madagascar, Province Diego Suarez	CONN
Marchantia	*polymorpha*	Marchantiaceae	Y. Liu 20150001	USA, Connecticut	CONN
Marchantia	*polymorpha* *	Marchantiaceae	Deyholos		
Mniodendron	*camptotheca*	Hypnodendraceae	B. Shaw 16618	New Caledonia, Southern Province	DUKE/CONN
Mniodendron	*comosum*	Hypnodendraceae	Dalton August 2012	Australia, Marriotts Falls	CONN
Mnium	*hornum*	Mniaceae	Goffinet 11171	USA, Connecticut	CONN
Neckeropsis	*lepineana*	Neckeraceae	Goffinet 11938	Madagascar, Province Diego Suarez	CONN
Oedipodiella	*australis*	Gigaspermaceae	Goffinet 11561	South Africa, Eastern Cape Province	CONN
Oedipodium	*griffithianum*	Oedipodiaceae	Williston 8610	Canada, British Columbia	CONN
Orthodontium	*lineare*	Orthodontiaceae	Buck 60455	Chile, Antártica Chilena	NY
Orthorrhynchium	*elegans* 1	Orthorrhynchiaceae	Shevock 45053	Philippines, North Cotabato	CONN
Orthorrhynchium	*elegans* 2	Orthorrhynchiaceae	Shevock 44162	New Zealand, Nelson-Marlborough	CONN
Orthotrichum	*stellatum*	Orthotrichaceae	Goffinet 10579	USA, Connecticut	CONN
Philonotis	*vagans*	Bartramiaceae	Goffinet 11059	Chile, Antártica Chilena	CONN
Physcomitrella	*patens*	Funariaceae	Spribille January 02	Canada, British Columbia	CONN

Pilotrichum	*evanescens*	Pilotrichaceae	Lavocat 15406	Guadeloupe	CONN
Plagiothecium	*laetum*	Plagiotheciaceae	J. Shaw 17142	USA, North Carolina	DUKE
Pogonatum	*convolutum*	Polytrichaceae	Goffinet 12308	Madagascar, Diego Suarez	CONN
Pohlia	*cruda*	Mniaceae	Goffinet 11057	Chile, Antártica Chilena	CONN
Pseudotaxiphyllum	*elegans*	Plagiotheciaceae	Carter 7598	USA, California	DUKE
Pterobryella	*rigida*	Pterobryellaceae	Bell 04.11.08.006	New Caledonia	H
Ptilidium	*pulcherrimum*[*]	Ptilidiaceae	Larsson AL602		UPS
Ptychomitrium	*cucullatifolium*	Ptychomitriaceae	Goffinet 10224	South Africa, Western Cape	CONN
Ptychomnion	*cygnisetum*	Ptychomniaceae	Buck 58936	Chile, Antártica Chilena	NY/CONN
Pulchrinodus	*inflatus*	Pulchrinodaceae	Shevock 44431	New Zealand, Westland	CONN
Pyrrhobryum	*spiniforme*	Rhizogoniaceae	Goffinet 11631	South Africa, Western Cape	CONN
Racopilum	*sp.*	Racopilaceae	Goffinet 11885	Madagascar, Diego Suarez	CONN
Rhabdodontium	*buftonii*	Pterobryaceae	Dalton s.n.	Australia, Tasmania	CONN
Rhabdoweisia	*crispata*	Rhabdoweisiaceae	Goffinet 11140	USA, North Carolina	CONN
Rhacocarpus	*purpurascens*	Rhacocarpaceae	Goffinet & Cox 7303	Chile, Los Lagos	CONN
Rhizogonium	*distichum*	Rhizogoniaceae	Dalton s.n.	Australia, Marriotts Falls	CONN
Rhodobryum	*ontariense*	Bryaceae	Goffinet 11152	USA, North Carolina	CONN
Scapania	*nemorea*	Scapaniaceae	Goffinet 10589	USA, Connecticut	CONN
Scapania	*nemorosa*[*]	Scapaniaceae	Pokorny NC19	USA, North Carolina	DUKE
Schimperobryum	*splendidissimum*	Schimperobryaceae	Goffinet 12841	Chile, Los Lagos	CONN
Schlotheimia	*ferruginea*	Orthotrichaceae	Goffinet 10318	South Africa, Western Cape	CONN
Sciadocladus	*menziesii*	Pterobryellaceae	B. Shaw 16562	New Caledonia, Southern Province	DUKE/CONN
Scouleria	*aquatica*	Scouleriaceae	Shevock 41844	USA, Oregon	CONN
Sphagnum	*girgensohnii*	Sphagnaceae	Goffinet 10588	USA, Connecticut	CONN
Sphagnum	*lescurii*[*]	Sphagnaceae			
Sphagnum	*palustre* 1[*]	Sphagnaceae	Rothfels 4143	USA, North Carolina	DUKE
Sphagnum	*palustre* 2	Sphagnaceae	Goffinet 10575	USA, Connecticut	CONN

Sphagnum	*recurvum*[*]	Sphagnaceae	Rothfels 4144	USA, North Carolina	DUKE
Spiridens	*camusii*	Hypnodendraceae	B. Shaw 16807	New Caledonia, Southern Province	DUKE/CONN
Symphysodontella	*subulata*	Pterobryaceae	Shevock 44844	Philippines, Bukidnon	CONN
Syntrichia	*anderssonii*	Pottiaceae	Goffinet 11056	Chile, Antártica Chilena	CONN
Takakia	*ceratophylla*	Takakiaceae	Ma 13-5064	China, Yunnan	CONN
Takakia	*lepidozioides* 1[*]	Takakiaceae	Chang 05-02	Canada, British Columbia	UBC
Takakia	*lepidozioides* 2	Takakiaceae	Sears May 3, 2015	Canada, British Columbia	CONN
Taxithelium	*planum*	Pylaisiadelphaceae	Lavocat 15407	Guadeloupe	CONN
Tayloria	*mirabilis*	Splachnaceae	Goffinet 11051	Chile, Antártica Chilena	CONN
Tetraphis	*pellucida* 1	Tetraphidaceae	Goffinet February 3, 2012	USA, Connecticut	CONN
Tetraphis	*pellucida* 2[*]	Tetraphidaceae	Larsson AL605		UPS
Tetrodontium	*brownianum*	Tetraphidaceae	Nebel 132182	Germany, Baden-Württemberg	STU
Thamnobryum	*hispidum*	Neckeraceae	Shevock 44212	New Zealand, Nelson-Marlborough	CONN
Thamnobryum	*pandum*	Neckeraceae	B. Shaw 17152	New Caledonia, Northern Province	CONN
Timmia	*norvegica*	Timmiaceae	Hedenäs June 13, 2013	Norway, Nordland	CONN
Timmia	*austriaca*	Timmiaceae	Hedenäs et al. September 11, 2013	Sweden, Ångermanland	CONN
Timmia	*megapolitana*	Timmiaceae	Budke 203	USA, New York	CONN
Timmia	*megapolitana*	Timmiaceae	Budke 202	USA, New York	CONN
Tortella	*humilis*	Pottiaceae	Goffinet 11158	USA, North Carolina	CONN
Touwiodendron	*diversifolium*	Hypnodendraceae	Bell 30.07.07.011	Malaysia, Sabah	H
Trachypodopsis	*serrulata*	Meteoriaceae	Goffinet 12373	Madagascar, Diego Suarez	CONN
Ulota	*hutchinsiae*	Orthotrichaceae	Goffinet 10580	USA, Connecticut	CONN
Yunnanobryon	*rhyacophilum*	Regmatodontaceae	Shevock 35406	China, Yunnan	CONN
Zygodon	*viridisssimus_v_rupestris*	Orthotrichaceae	Harpel 51552	USA, Washington	DUKE

Supplementary Table 2. Genome references for designing the plastid, mitochondrial, and nuclear baits.

Genome	Group	Taxon	Number of gene	Length (kb)	GenBank accession
Plastid	Liverworts	*Haplomitrium*	81	60	current study
		Marchantia	89	71	NC_001319
		Aneura	62	47	NC_010359
		Metzgeria	81	64	current study
		Ptilidium	80	59	NC_015402
	Mosses	*Physcomitrella*	83	70	NC_005087
		Syntrichia	81	65	NC_012052
		Anomodon	80	63	current study
		Hypnum	80	63	current study
	Hornworts	*Anthoceros*	86	70	NC_004543
		Nothoceros	82	66	NC_020259
	Lycophyte	*Huperzia*	87	74	NC_006861
	Fern	*Equisetum*	84	72	NC_014699
	Gymnosperm	*Cycas*	122	90	NC_009618
	Angiosperm	*Amborella*	84	79	NC_005086
Mitochondrial	Liverworts	*Treubia*	39	30	NC_016122
		Marchantia	42	33	NC_001660
		Pleurozia	41	33	NC_013444
	Mosses	*Physcomitrella*	39	32	NC_007945
		Anomodon	40	32	NC_016121
		Hypnum	40	32	NC_024516
		Climacium	40	32	NC_024515
	Hornworts	*Nothoceros*	21	17	NC_012651
		Phaeoceros	19	17	NC_013765
	Lycophyte	*Huperzia*	35	27	NC_017755
	Gymnosperm	*Cycas*	39	34	NC_010303
	Angiosperm	*Lotus*	34	31	NC_016743
Nuclear	Mosses	*Physcomitrella*			Rensing et al. (5)
		Anomodon			1KP Project (3)
		Hypnum			1KP Project (3)
		Climacium			1KP Project (3)

Supplementary Table 3. Average substitution rates in mitochondrial (Mt), plastid (Pt) and nuclear (Nu) genes of mosses, relative rates in average synonymous and non-synonymous substitution rates and average non-synonymous to synonymous substitution rate ratios in mosses, and seed plants.

	**Mt**	**Pt**	**Nu**
**Synonymous substitutions**			
Mosses dS tree score	2.782421	15.83359	20.49185
Relative rates			
Mosses	1	5.7	7.4
Angiosperms*	1	3.1	10.0
Gymnosperms*	1	2.1	3.3
Seed plants*	1	2.7	6.0
**Non-synonymous substitutions**			
Mosses dN tree score	0.816526	1.053214	3.431693
Relative rates			
Mosses	1	1.3	4.2
Angiosperms*	1	2.5	3.4
Gymnosperms*	1	1.3	0.8
Seed plants*	1	1.8	1.7
**dN/dS ratio**			
Mosses	0.29	0.07	0.17
Angiosperms*	0.16	0.12	0.05
Gymnosperms*	0.25	0.15	0.06
Seed plants*	0.20	0.14	0.05

*Measurements are from Drouin et al. (6), based on the LWL85 model.

Supplementary Table 4. Phylogenic analyses performed for plastid, mitochondrial and nuclear genome data. The numbers in the bracket indicate the supplemental figure's number**.**

Analysis	Data	Program	Plastid	Mitochondrial	Organellar	Nuclear
Concatenation	nt	RAxML	100 BS, 3 partitions, GTR+G (**7**)	100 BS, 3 partitions , GTR+G (**11**)	100 BS, 6 partitions , GTR+G (**15**)	100 BS, 3 partitions , GTR+G (**18**)
		MrBayes	5M, 3 partitions (**8**)	5M, 3 partitions (**12**)	5M, 6 partitions (**16**)	5M, 3 partitions (**19**)
	aa	RAxML	300 BS, 16 partitions, gcpREV+G+F (**9**)	300 BS, 8 partitions, stmtREV+G+F (**13**)	100 BS, 24 partitions (**17**)	300 BS, 93 partitions (**20**)
		Phylobayes	CAT model (**10**)	CAT model (**14**)		CAT model (**21**)
Coalescent	nt	ASTRAL				RAXML gene trees, LPP (**22**)
		ASTRAL				Multi-locus bootstrapping (**23**)
	aa	ASTRAL				RAXML gene trees, LPP (**24**)
		ASTRAL				Multi-locus bootstrapping (**25**)
Concordant	nt	Phyparts				Congruence analysis, nt (**26**)
	aa	Phyparts				Congruence analysis, aa (**27**)

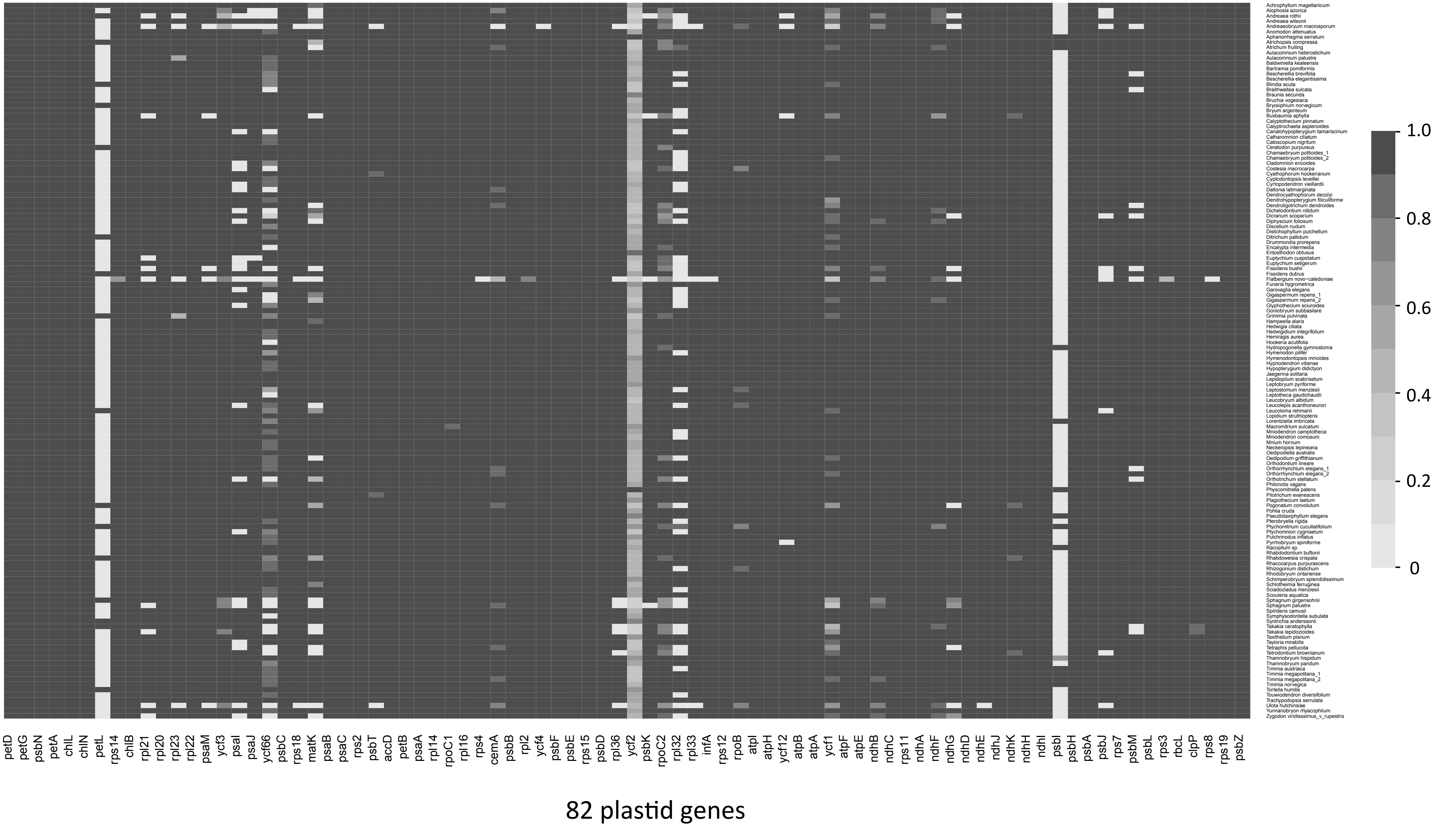

Supplementary Figure 1. Heat map showing the percent length of the enriched plastid genes recovered for 136 mosses. For *Eosphagnum inretortum, Ambuchanania leucobryoides, and Flatbergium sericeum*, transcriptome data were used to extract the plastid genes, so the three species were not included in the heat map.

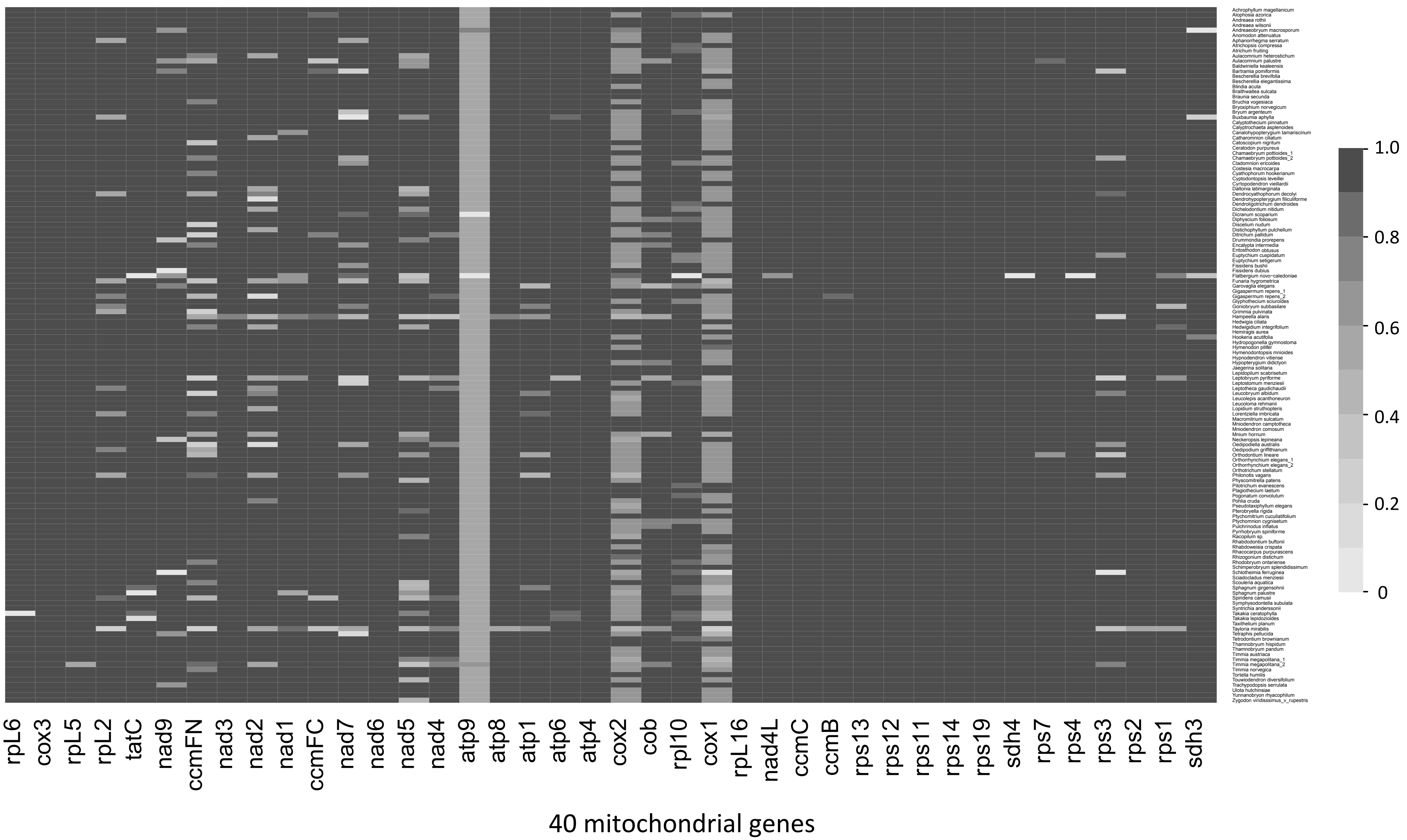

Supplementary Figure 2. Heat map showing the percent length of the enriched mitochondrial genes recovered for 136 mosses. For *Eosphagnum inretortum, Ambuchanania leucobryoides, and Flatbergium sericeum,* transcriptome data were used to extract the mitochondrial genes, so the three species were not included in the heat map.

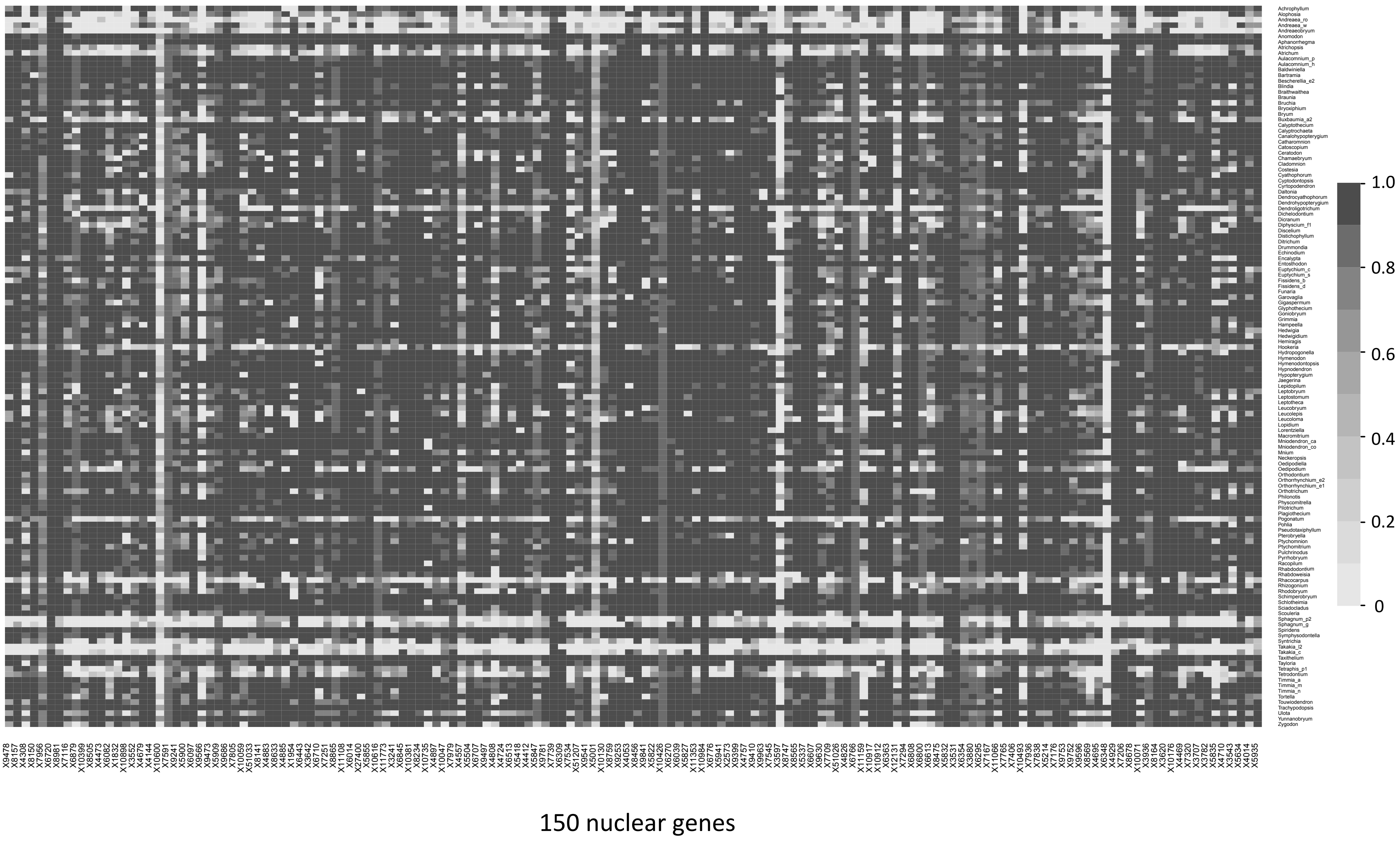

Supplementary Figure 3. Heat map showing the percent length of the enriched nuclear genes recovered for 130 mosses. Transcriptome data were used for extracting nuclear genes for the other mosses (See Supplementary Table 1 for details). The corresponding gene sequences can be found in the Dryad data database.

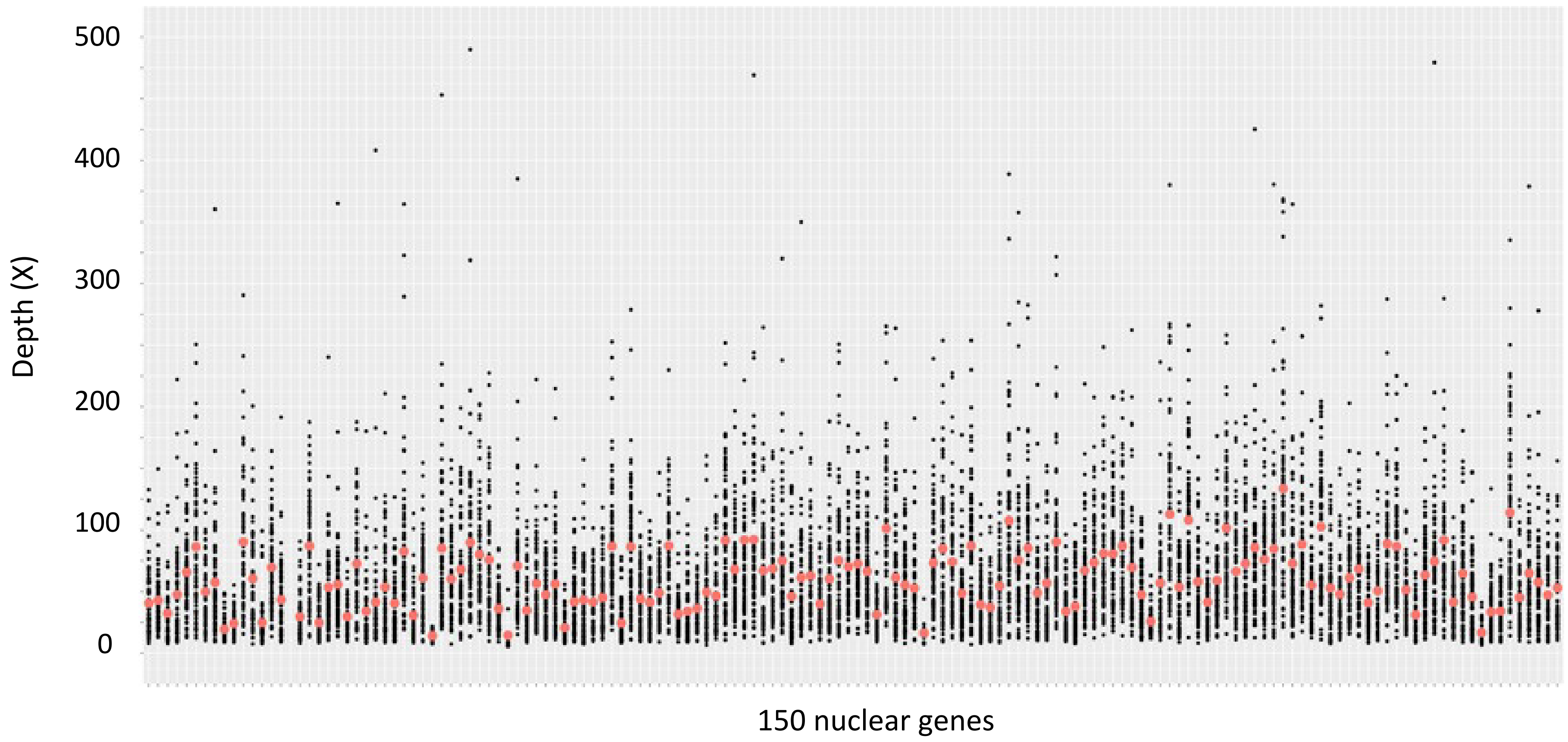

Supplementary Figure 4. Sequencing depth for the 150 nuclear genes in a set of the 96 pooled libraries. Each dot represents the depth of sequencing of one gene for one taxon. Red dots are the average depth for a given gene across all 96 taxa. Genes are shown in the same order as in Supplementary Figure 3.

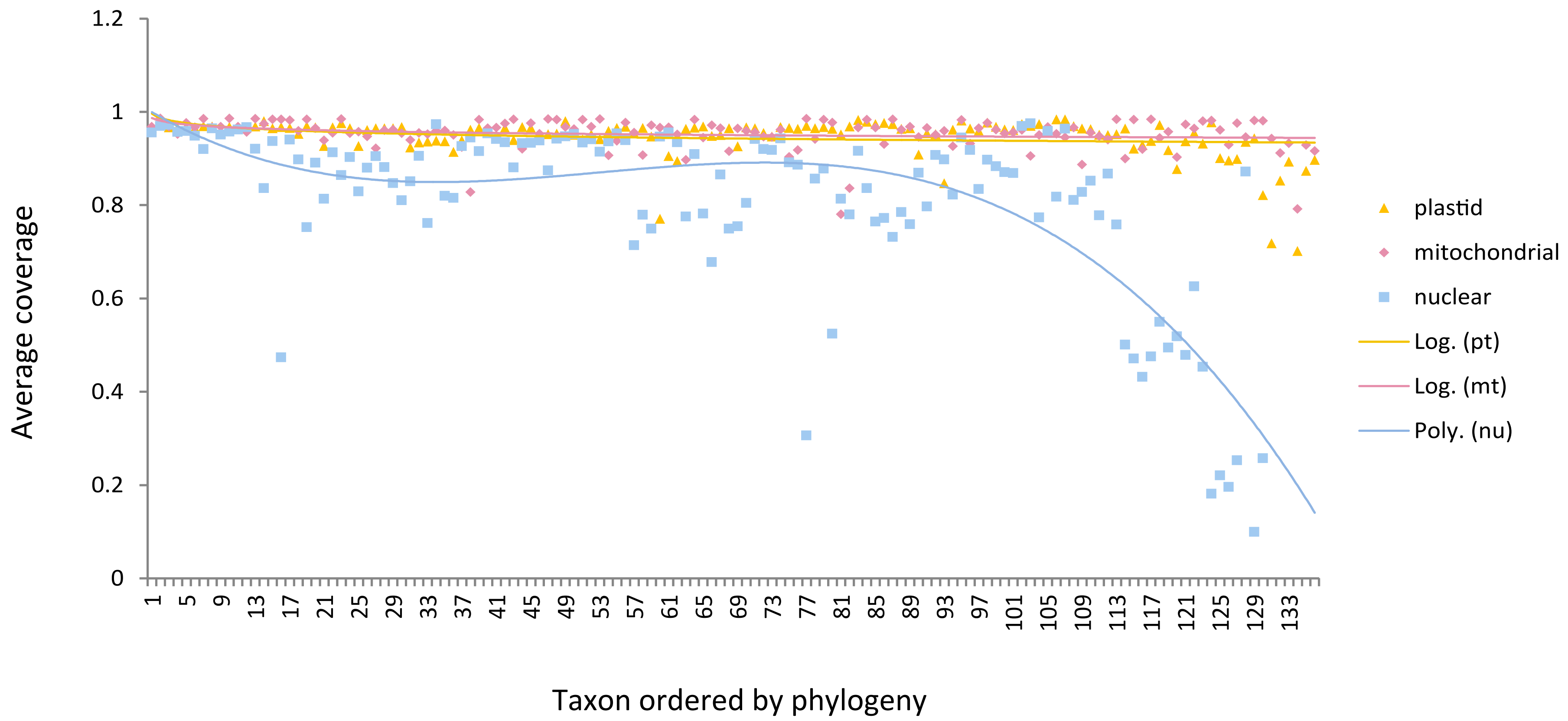

Supplementary Figure 5. Plot of the average percent length recovered for the targeted plastid, mitochondrial and nuclear genes of the sampled mosses. The taxa were sorted based on the phylogeny (See Figure 1A).

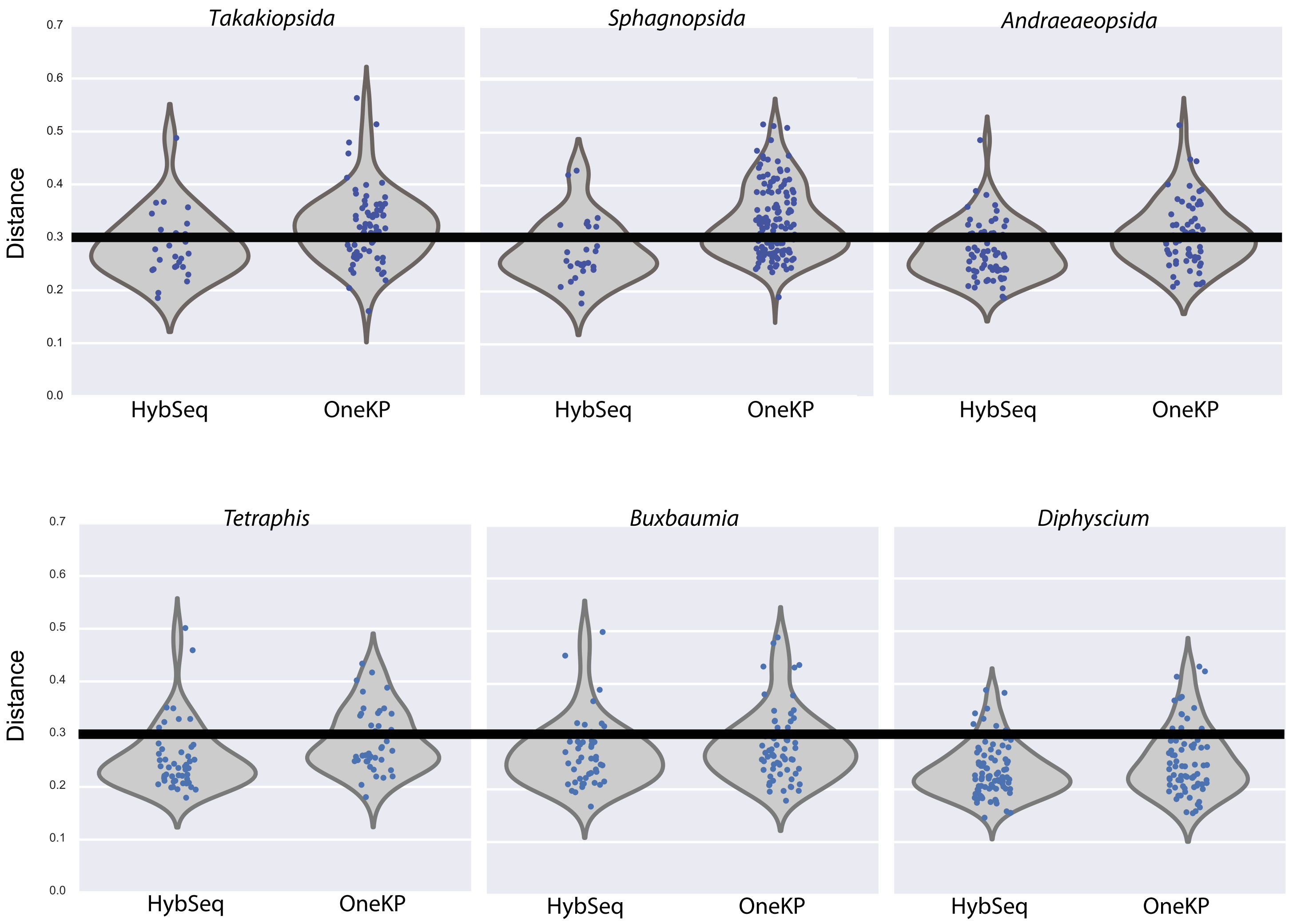

Supplementary Figure 6. Recovery efficiency of HybSeq for orders of mosses with high phylogenetic divergence from taxa used for probe design. Within each plot, each dot represents the pairwise divergence between the sequences used to design the probes (*Physcomitrella* and pleurocarpous mosses) a sequence recovered with HybSeq (left) or a transcriptome sequence assembled as part of OneKP (right). The transcripts serve as a reference of "known" sequence divergence; the line represents 30% sequence divergence, above which HybSeq recovery efficiency for the same genes is highly reduced.

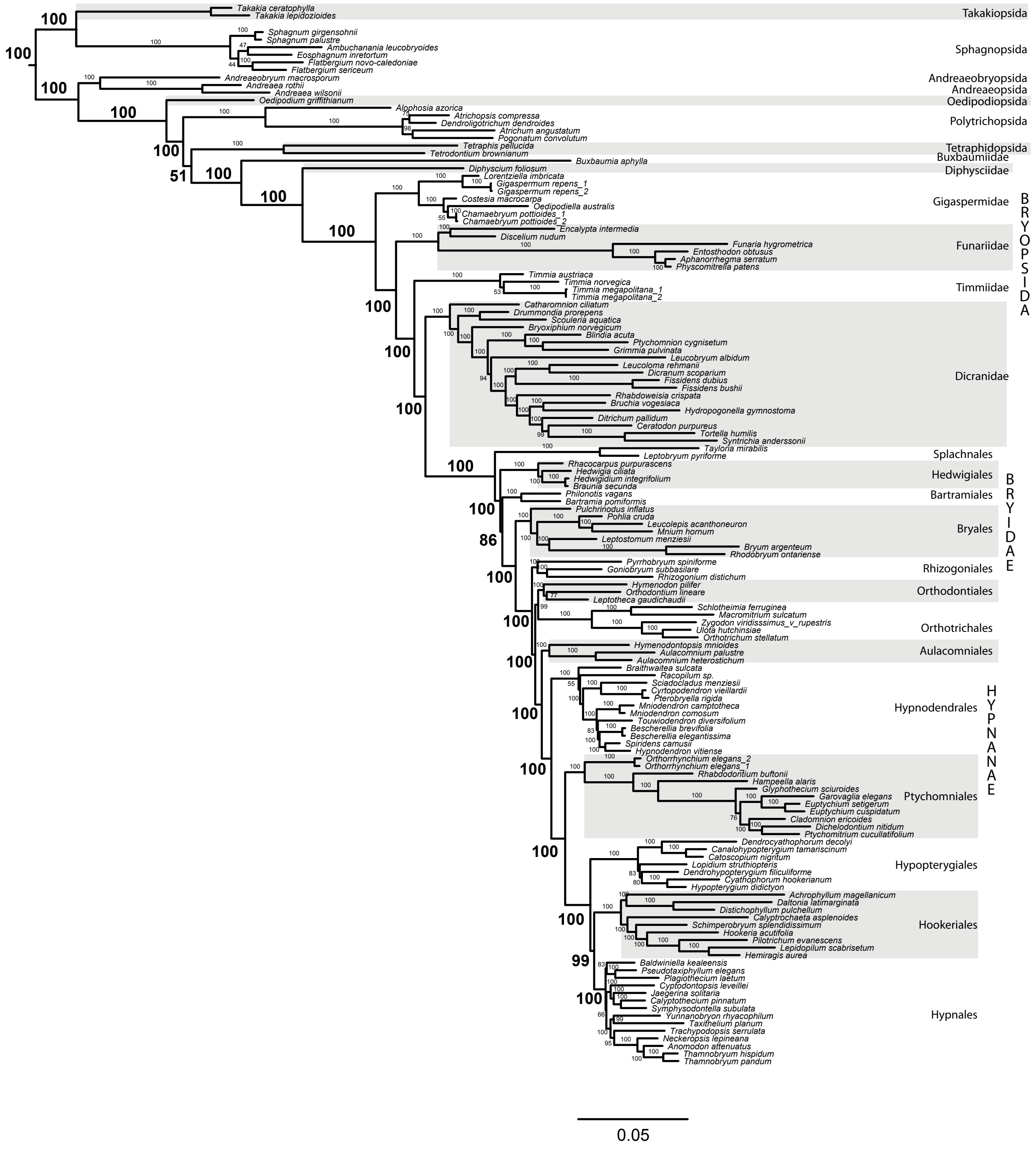

Supplementary Figure 7. Plastid genome DNA data: RAxML ML phylogram inferred from the plastid DNA data with GTR+G model; data partitioned in 1st, 2nd and 3rd codon positions, and 300 bootstrap replicates. Unit for scale bar is substitutions per site.

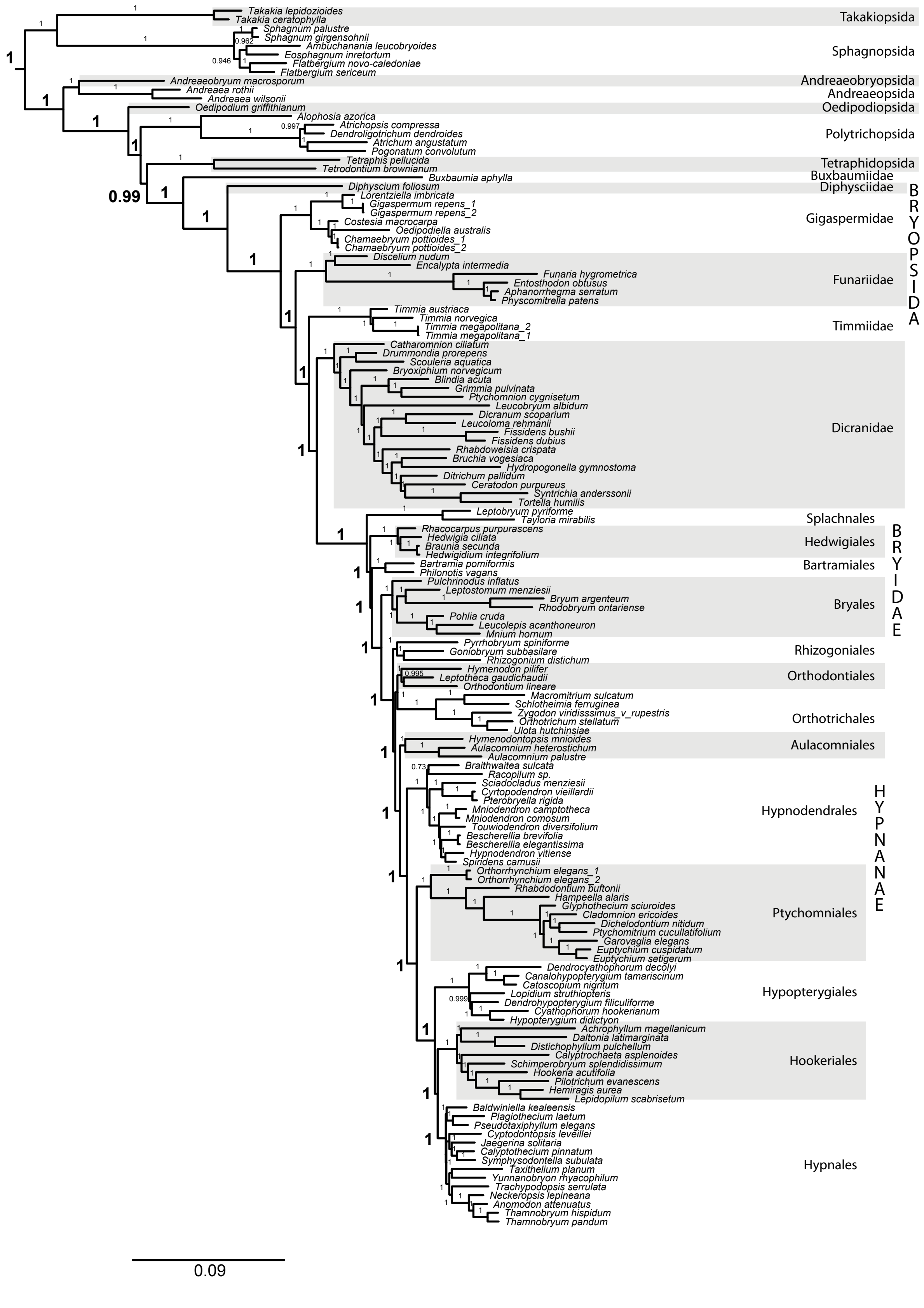

Supplementary Figure 8. Plastid genome DNA data: MCMC MrBayes codon site-specific 3*(GTR+G). Unit for scale bar is substitutions per site.

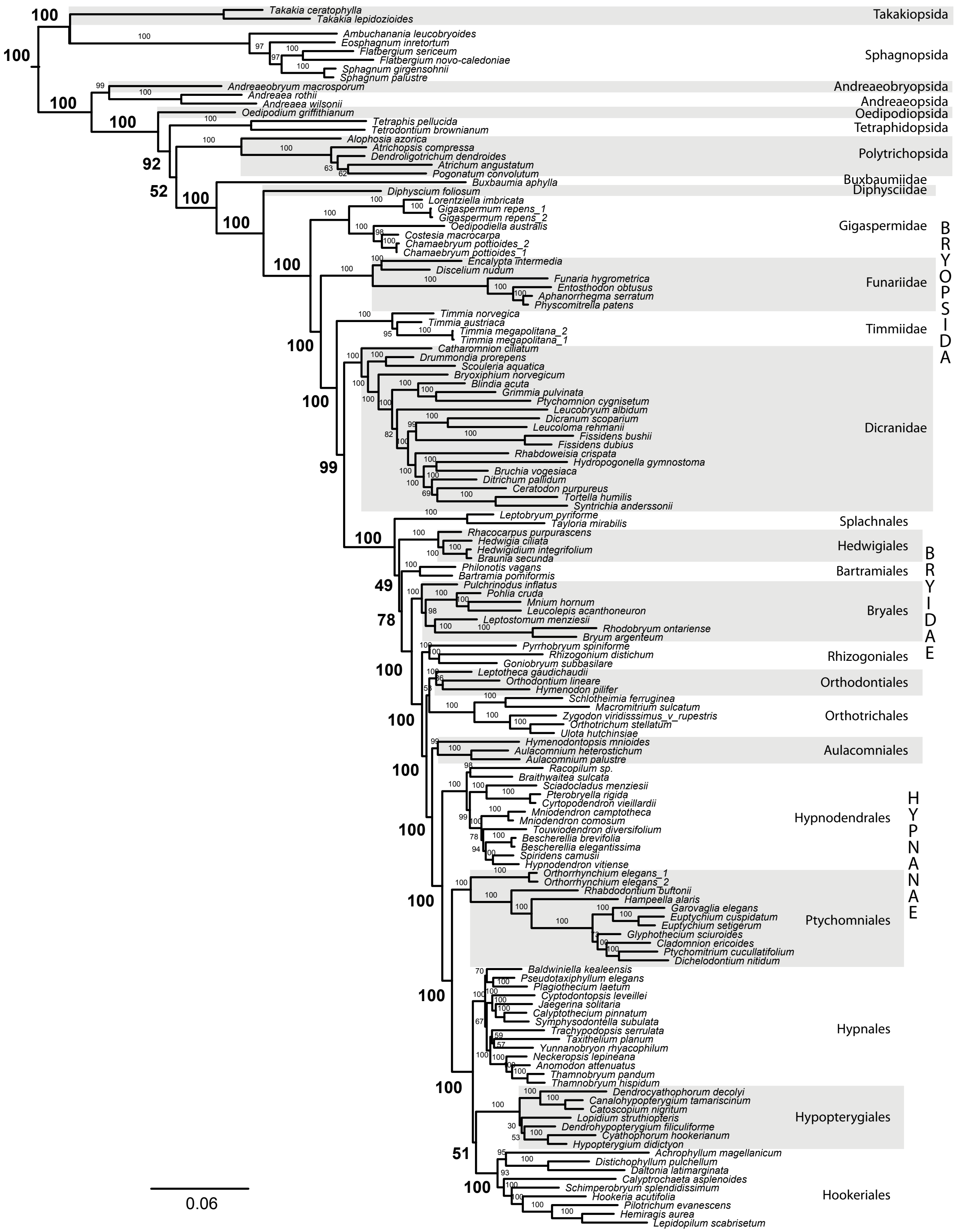

Supplementary Figure 9. Plastid genome AA data: RAxML ML phylogram inferred from the plastid AA data with gcpREV+G+F model, 16 data partitions, 300 bootstrap replicates. Unit for scale bar is substitutions per site.

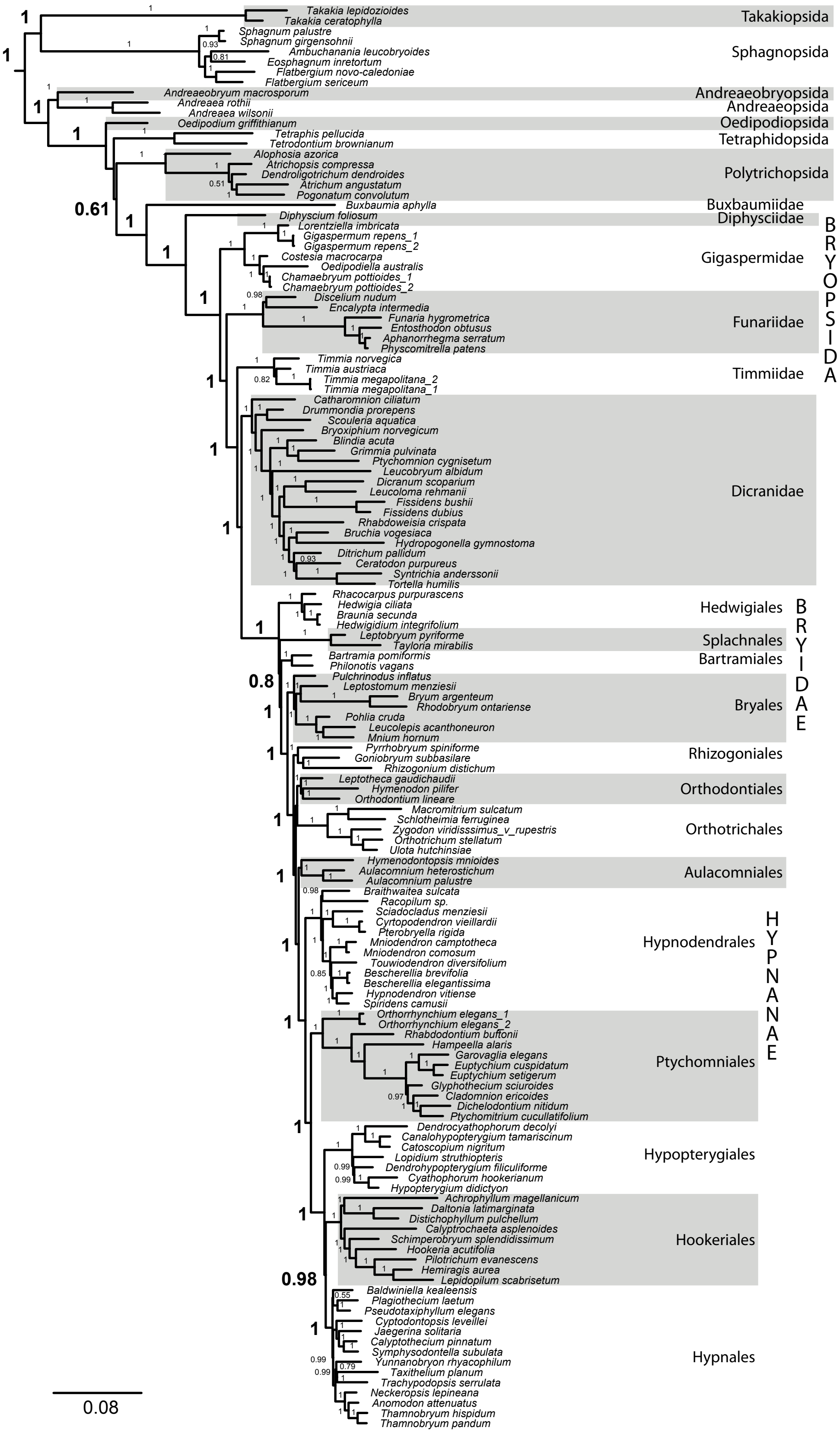

Supplementary Figure 10. Plastid AA data: MCMC Phylobayes tree with CAT+GTR+G model. Unit for scale bar is substitution per site.

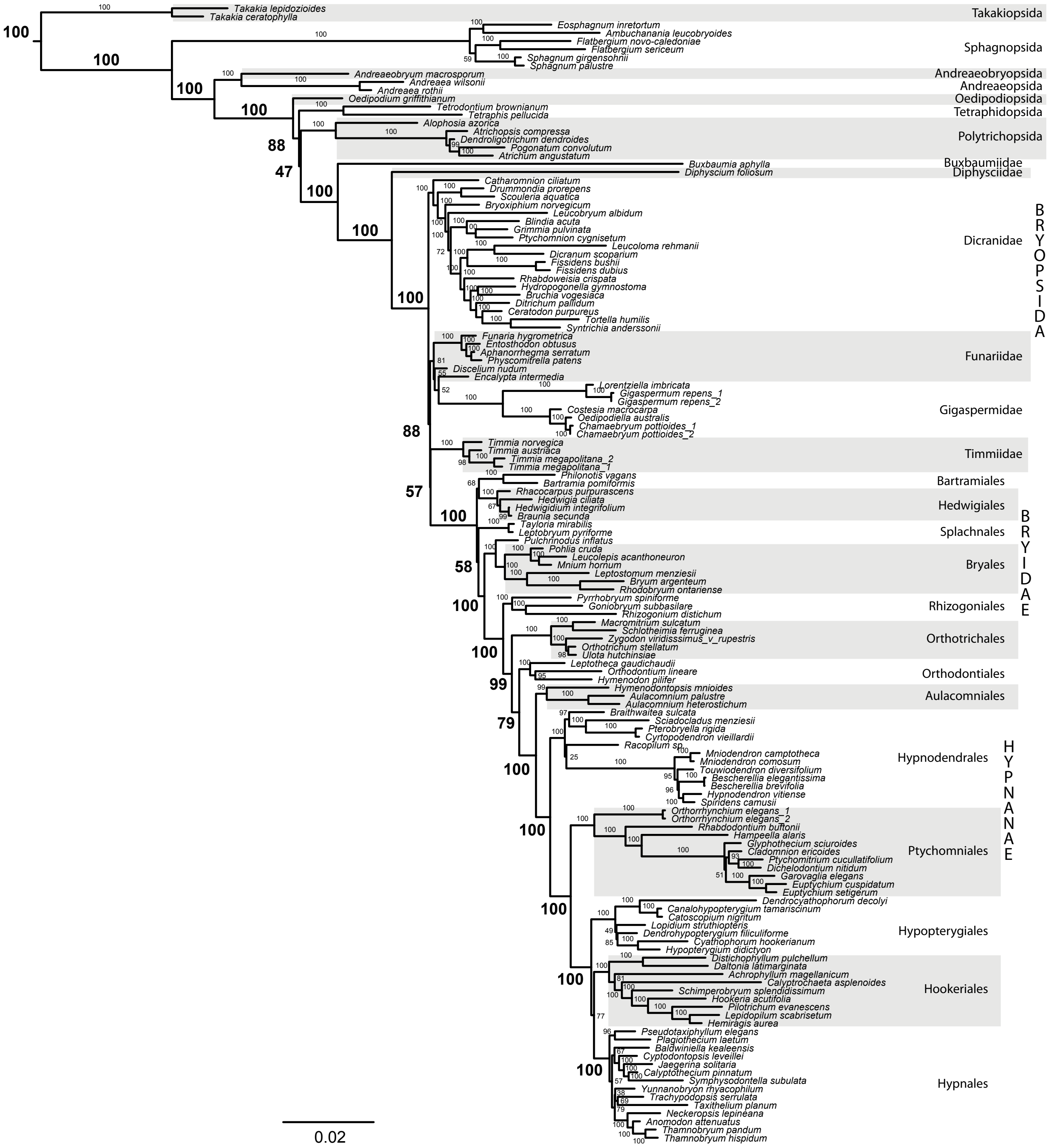

Supplementary Figure 11. Mitochondrial genome DNA data: RAxML ML phylogram inferred from the mitochondrial DNA data with GTR+G model, data partitioned in 1st, 2nd and 3rd codon positions, 300 bootstrap replicates. Unit for scale bar is substitution per site.

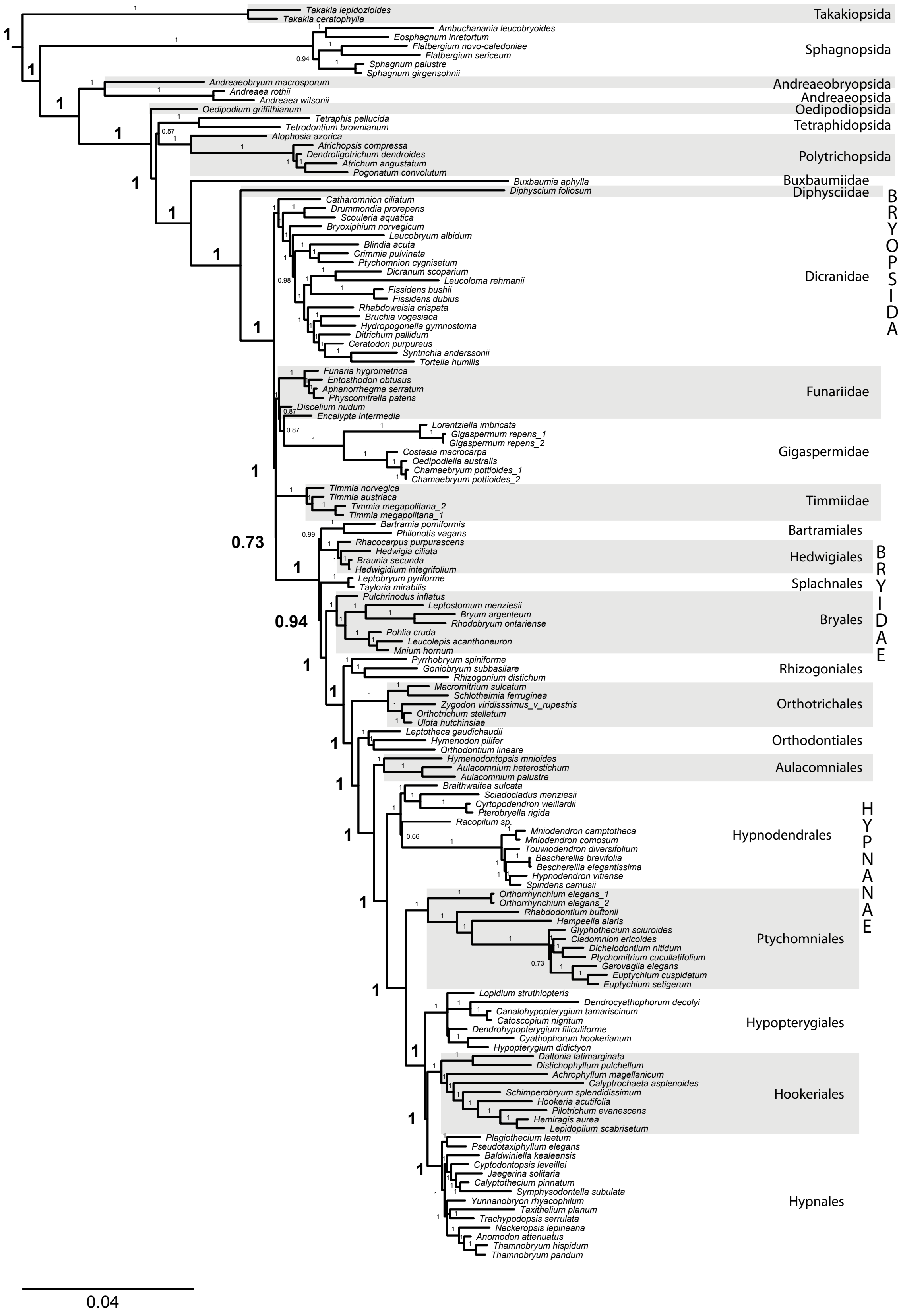

Supplementary Figure 12. Mitochondrial genome DNA data: MCMC MrBayes codon site-specific 3*(GTR+G). Unit for scale bar is substitution per site.

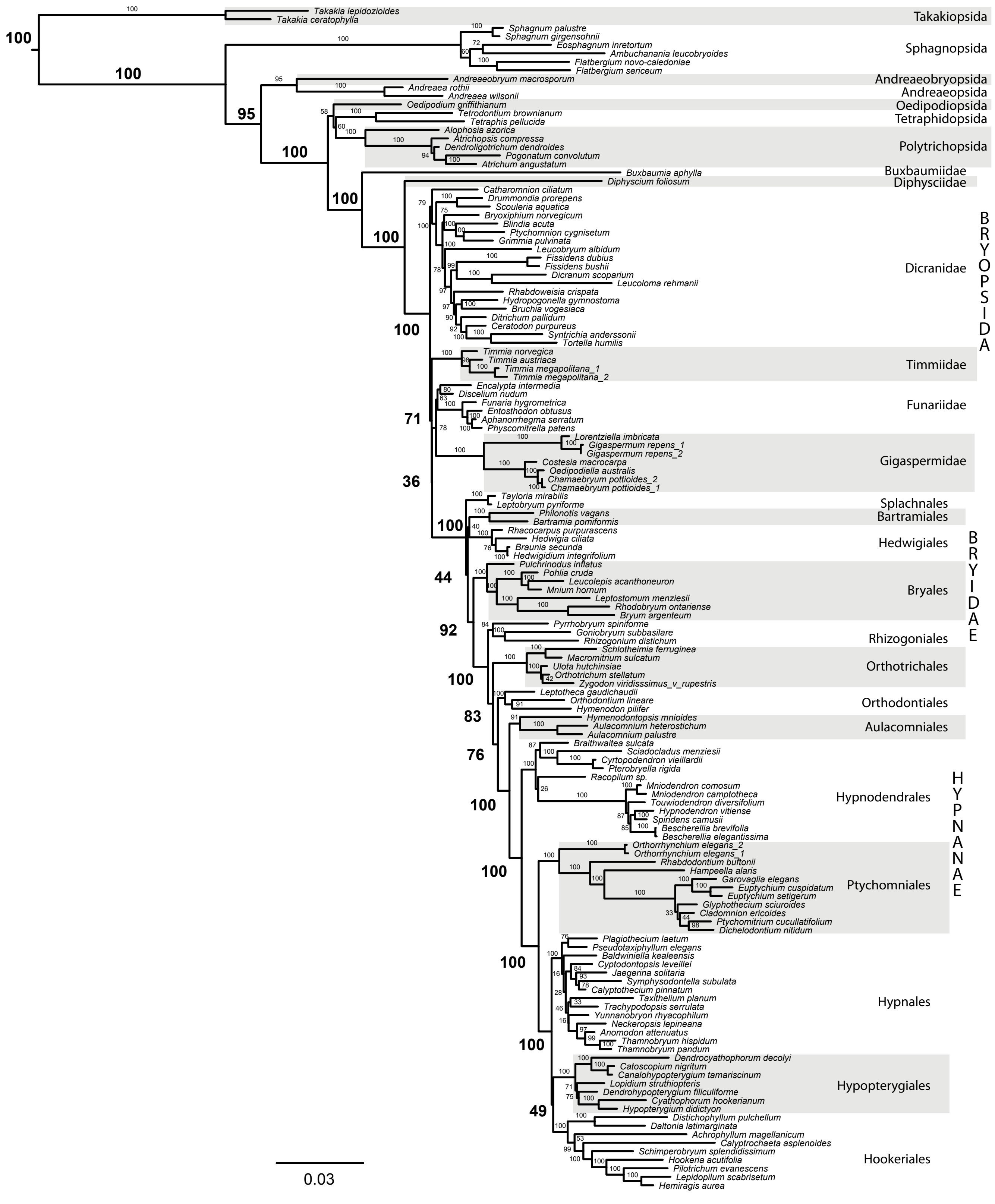

Supplementary Figure 13. Mitochondrial genome AA data: RAxML ML phylogram inferred from the mitochondrial AA data, with stmtREV+G+F model, eight data partitions, 300 bootstrap replicates. Unit for scale bar is substitution per site.

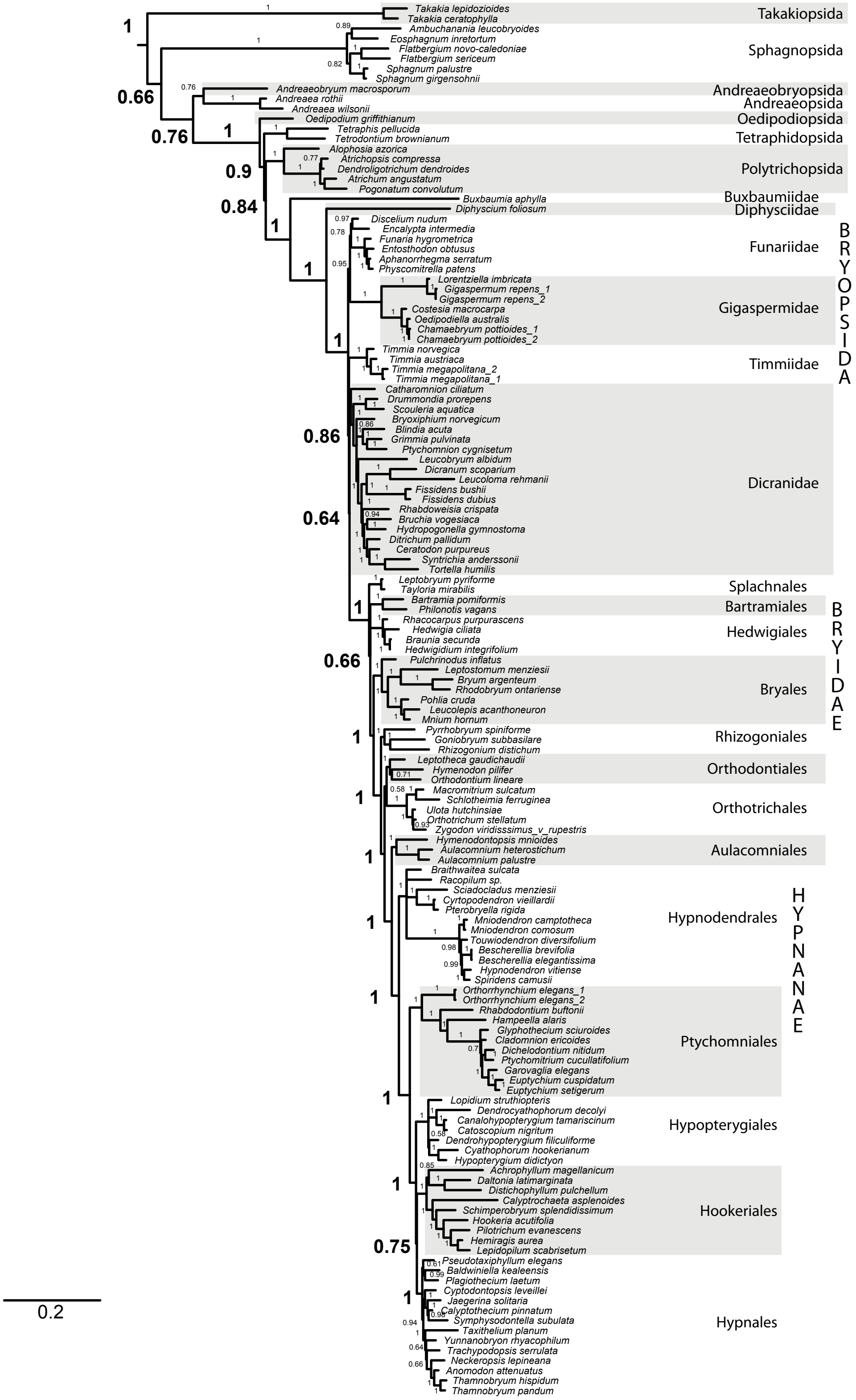

Supplementary Figure 14. Mitochondrial genome AA data: MCMC phylobayes analysis with CAT +GTR+G model. Unit for scale bar is substitution per site.

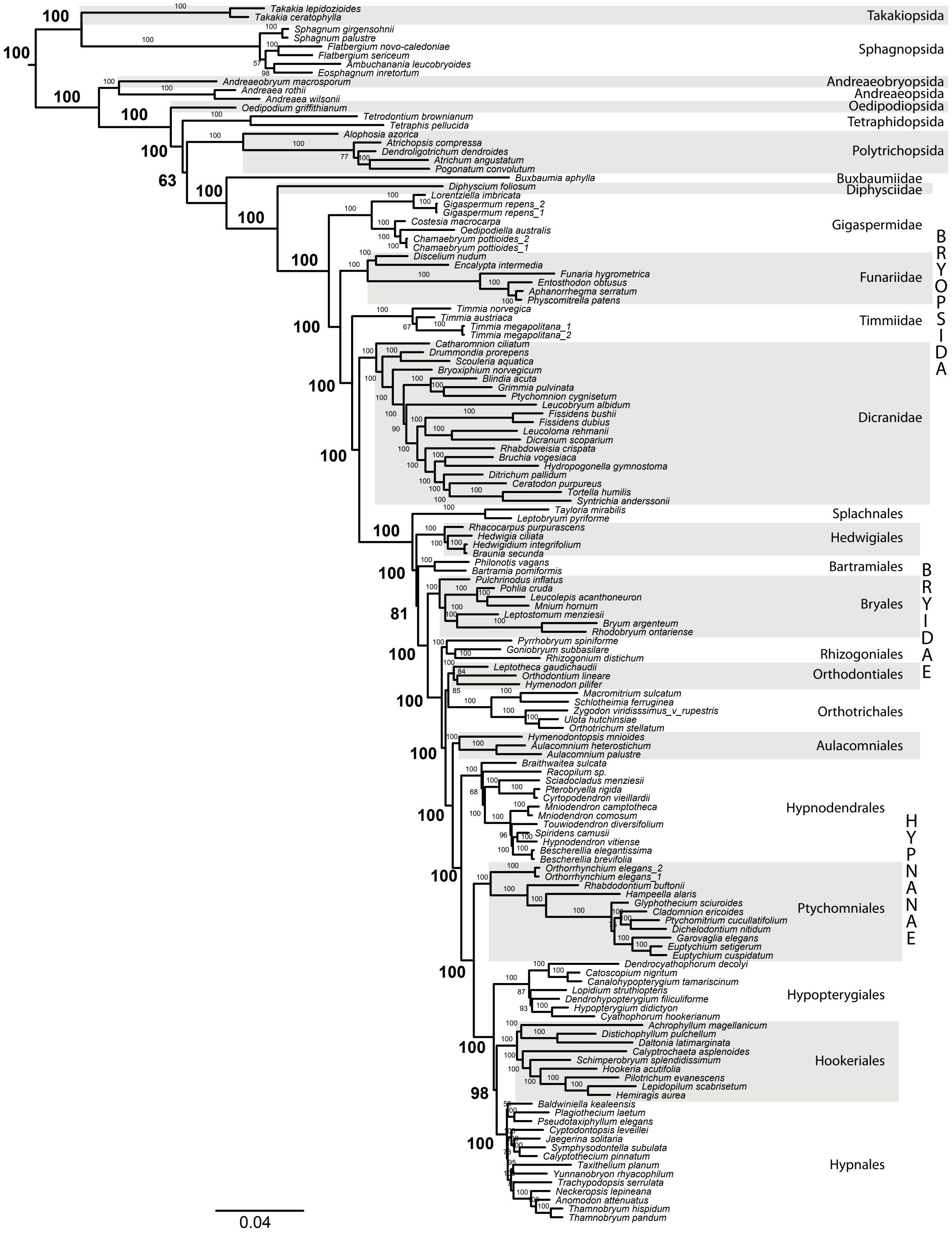

Supplementary Figure 15. Organellar genome DNA data: RAxML ML phylogram inferred from the organellar DNA data, with GTR+G model, data 1st, 2nd and 3rd codoon positions, 300 bootstrap replicates .Unit for scale bar is substitution per site.

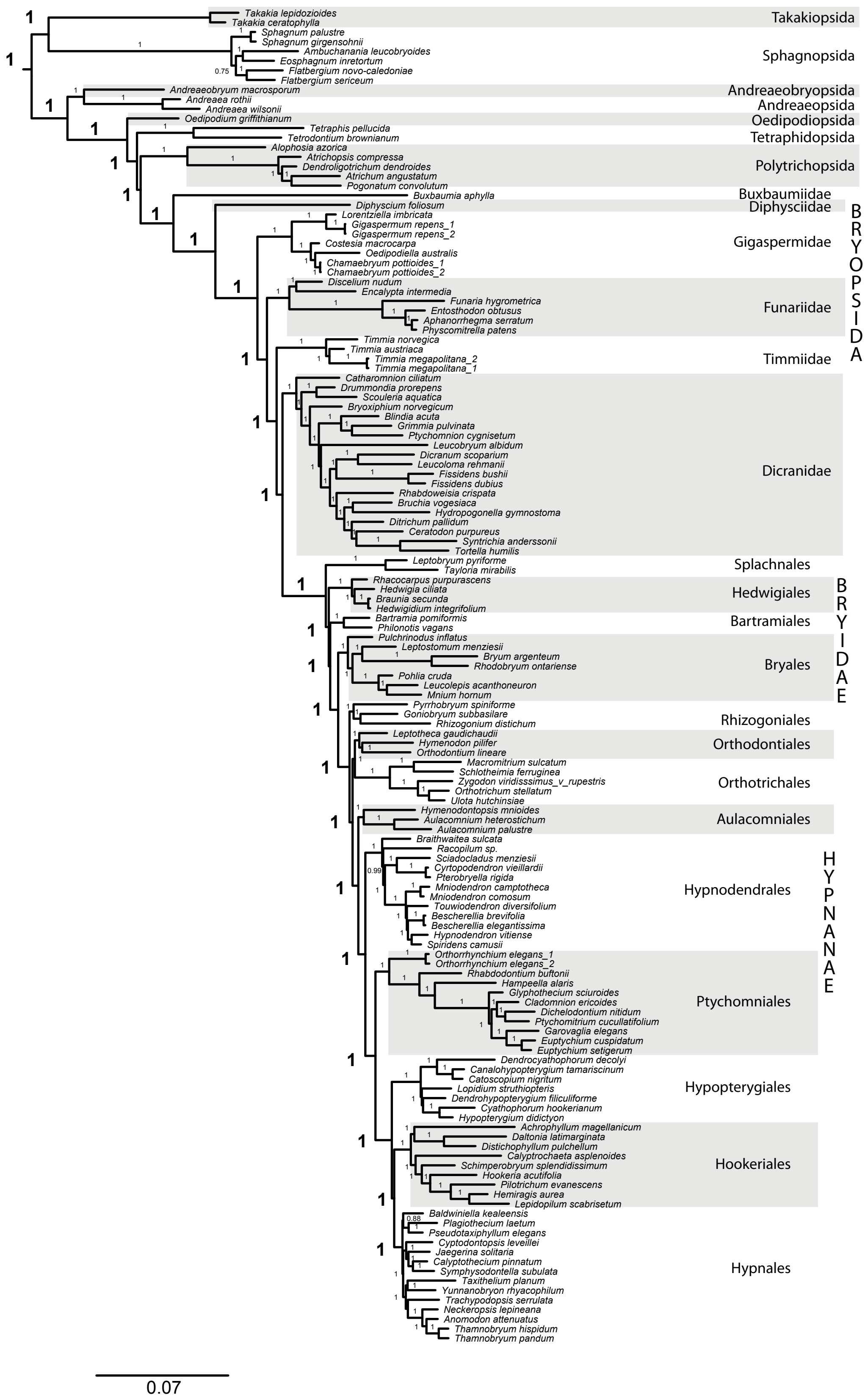

Supplementary Figure 16. Organellar genome DNA data: MCMC MrBayes codon plastid and mitochondrial site-specific 6*(GTR+G) model. Unit for scale bar is substitution per site.

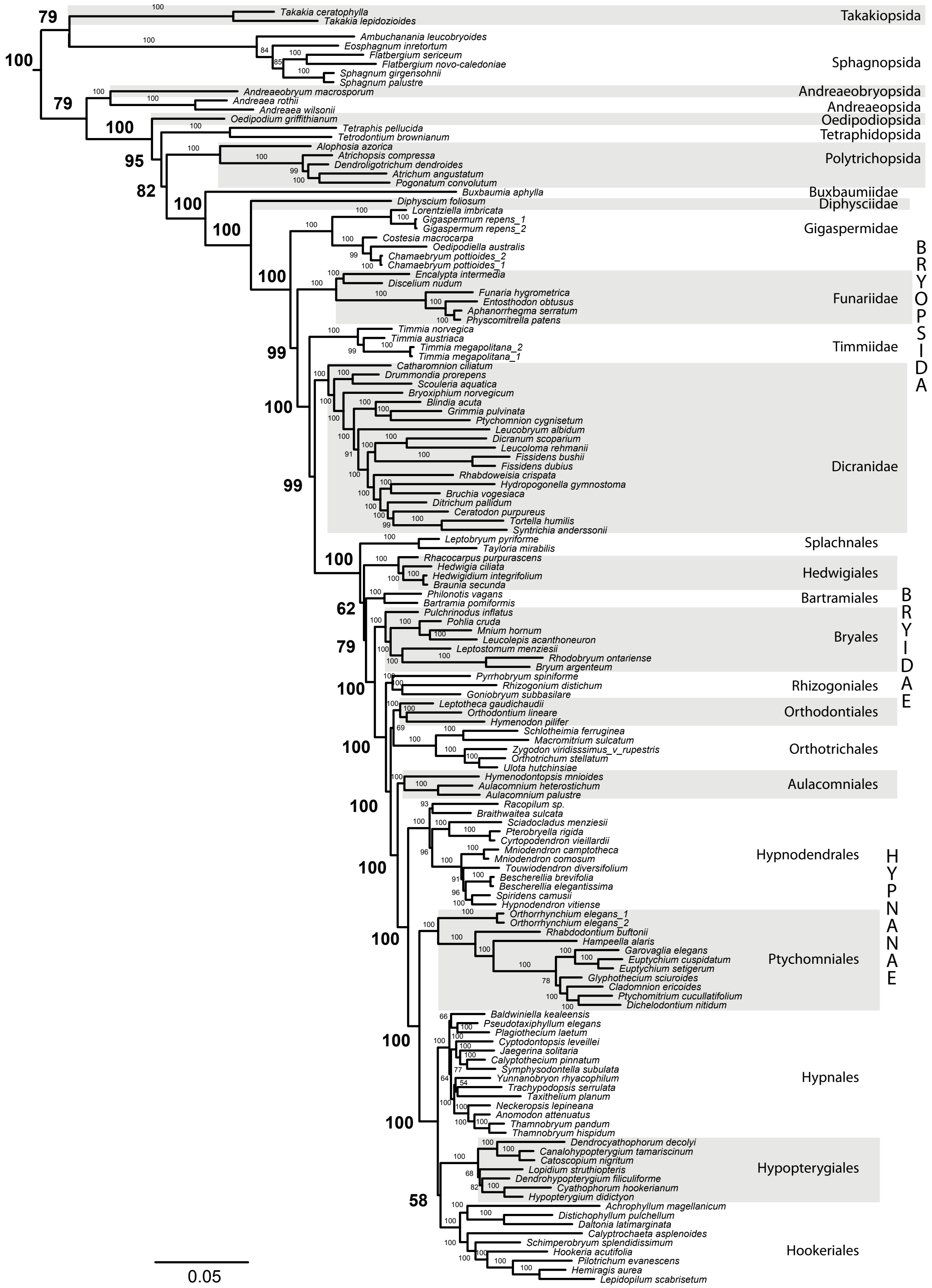

Supplementary Figure 17. Organellar genome AA data: RAxML ML phylogram inferred from the organellar AA data, with 8 data partitionos, 300 bootstrap replicates. Unit for scale bar is substitution per site.

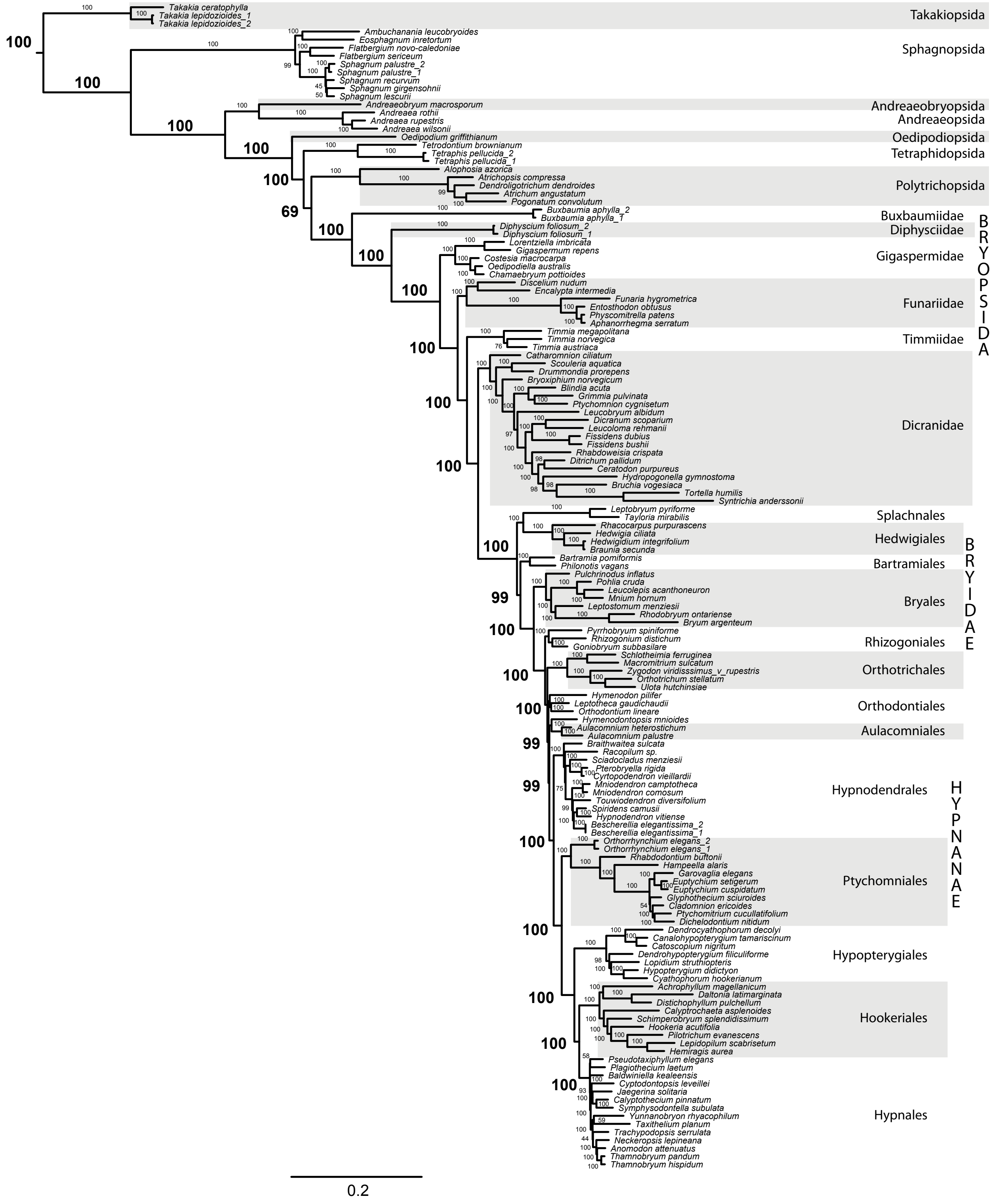

Supplementary Figure 18. Nuclear genome DNA data: RAxML ML phylogram inferred from the nuclear DNA data with GTR+G model, with data partitioned in 1st, 2nd and 3rd codon positions, 300 bootstrap replicates. Unit for scale bar is substitution per site.

.

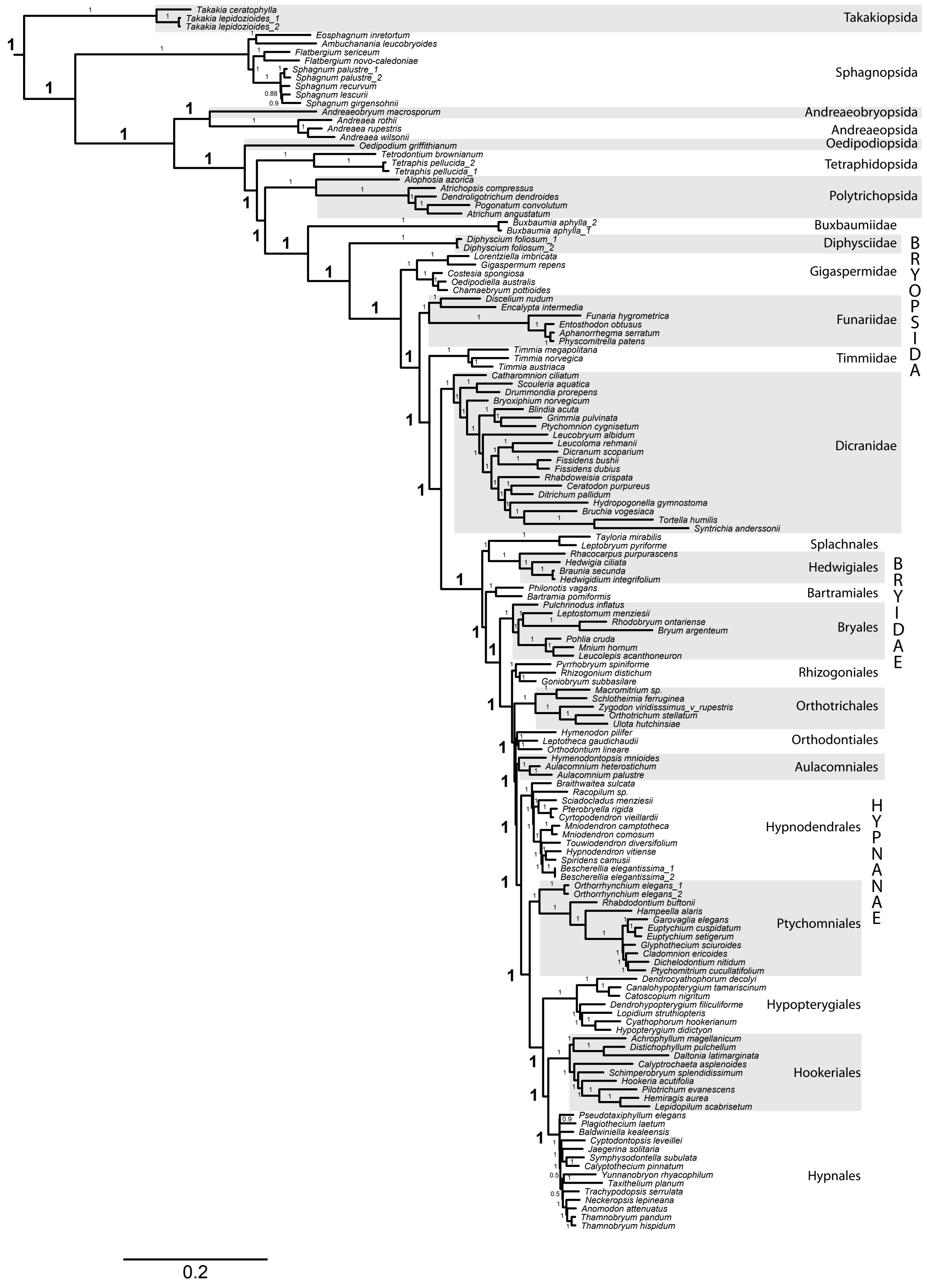

Supplementary Figure 19. Nuclear genome DNA data: MCMC MrBayes codon site-specific 3*(GTR+I+G). Unit in scale bar is substitution per site.

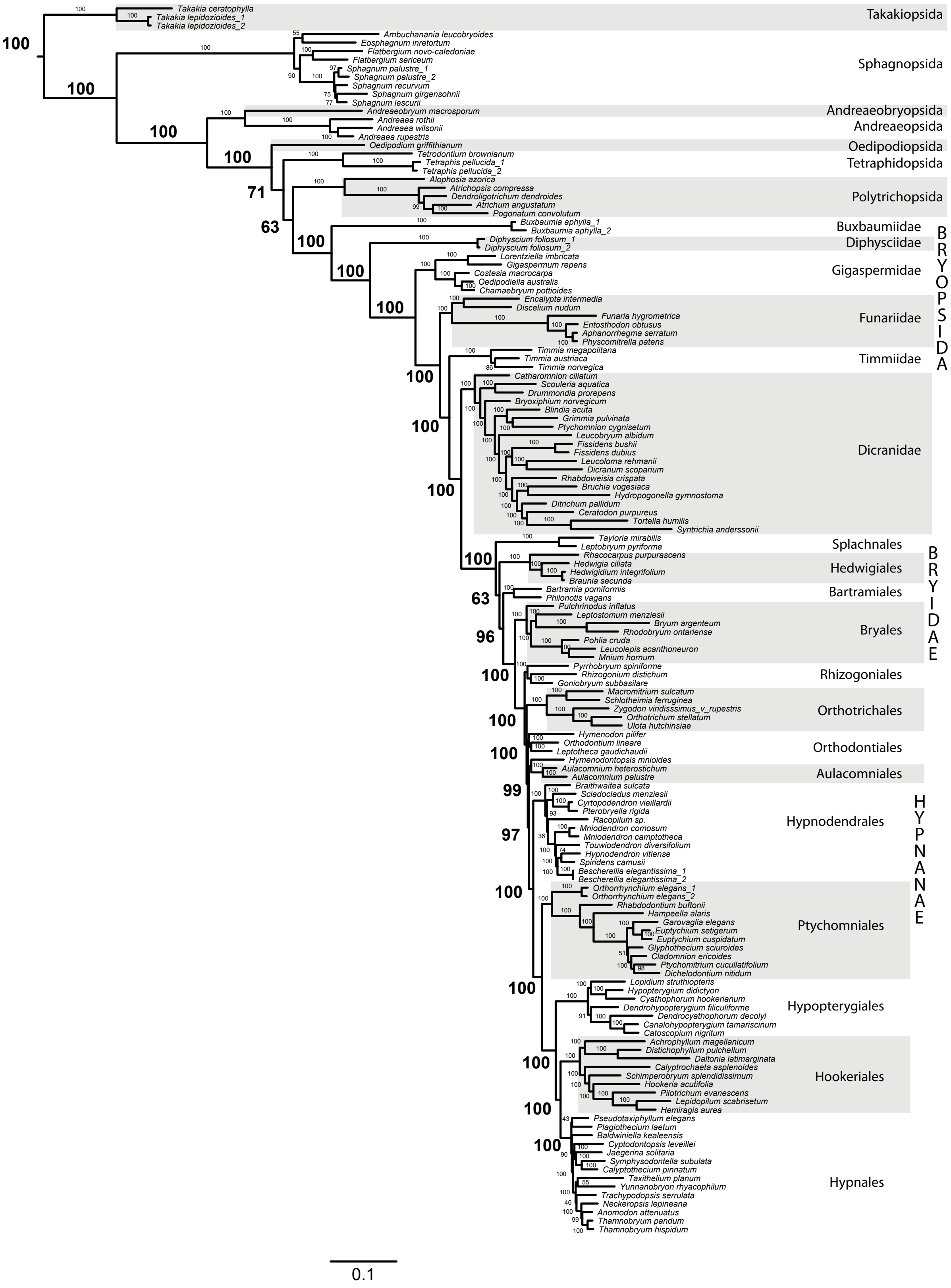

Supplementary Figure 20. Nuclear genome AA data: RAxML ML phylogram inferred from the nuclear AA data, with 93 data partitions, 300 bootstrap replicates. Unit is scale bar is substitution per site.

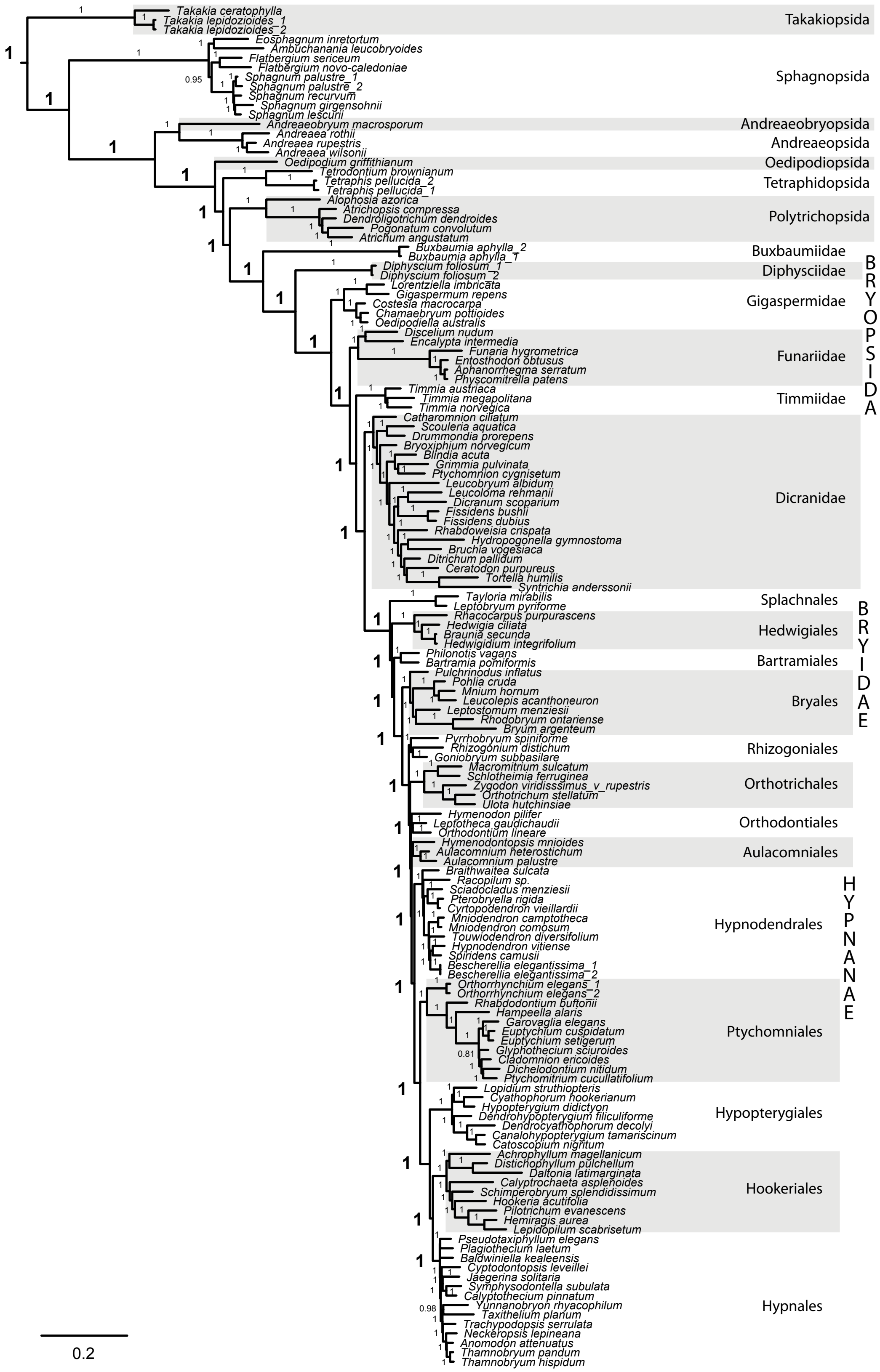

Supplementary Figure 21. Nuclear genome AA data: MCMC Phylobayes analysis with CAT+GTR +G model. Unit in scale bar is substitution per site.

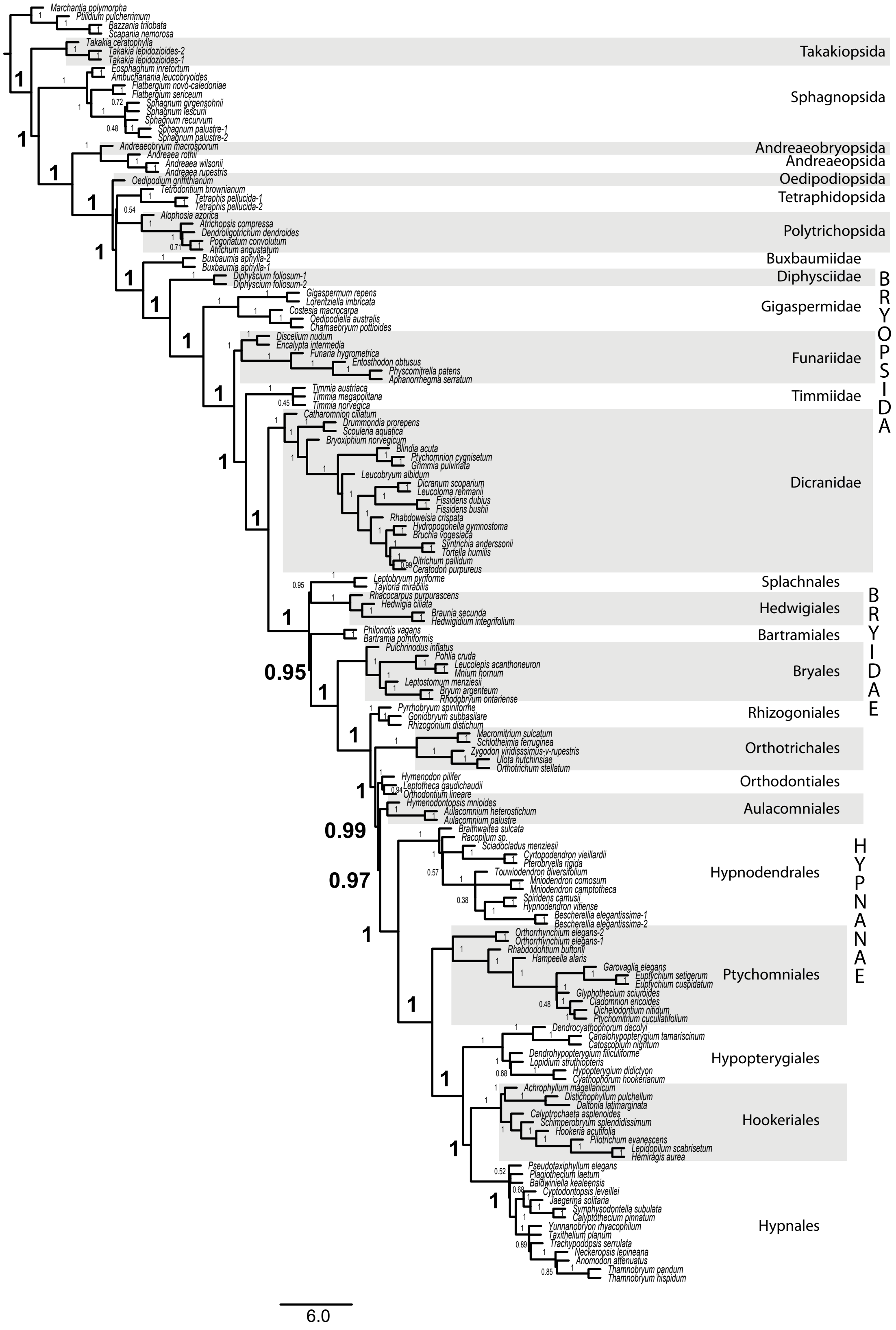

Supplementary Figure 22. Maximum quartet tree from ASTRAL, DNA trees, LPP Support: The tree is generated from 106 nucleoide RAXML gene trees. Prior to running ASTRAL, gene trees were collapsed to remove bipartitions not supported by at least 33% RAXML bootstrap support. Support values indicate the local posterior probability (LPP), which assesses the support for the quartet tree versus its three alternatives. Branch lengths in coalescent units (2*N generations) and are directly proportional to the amount of discordance.

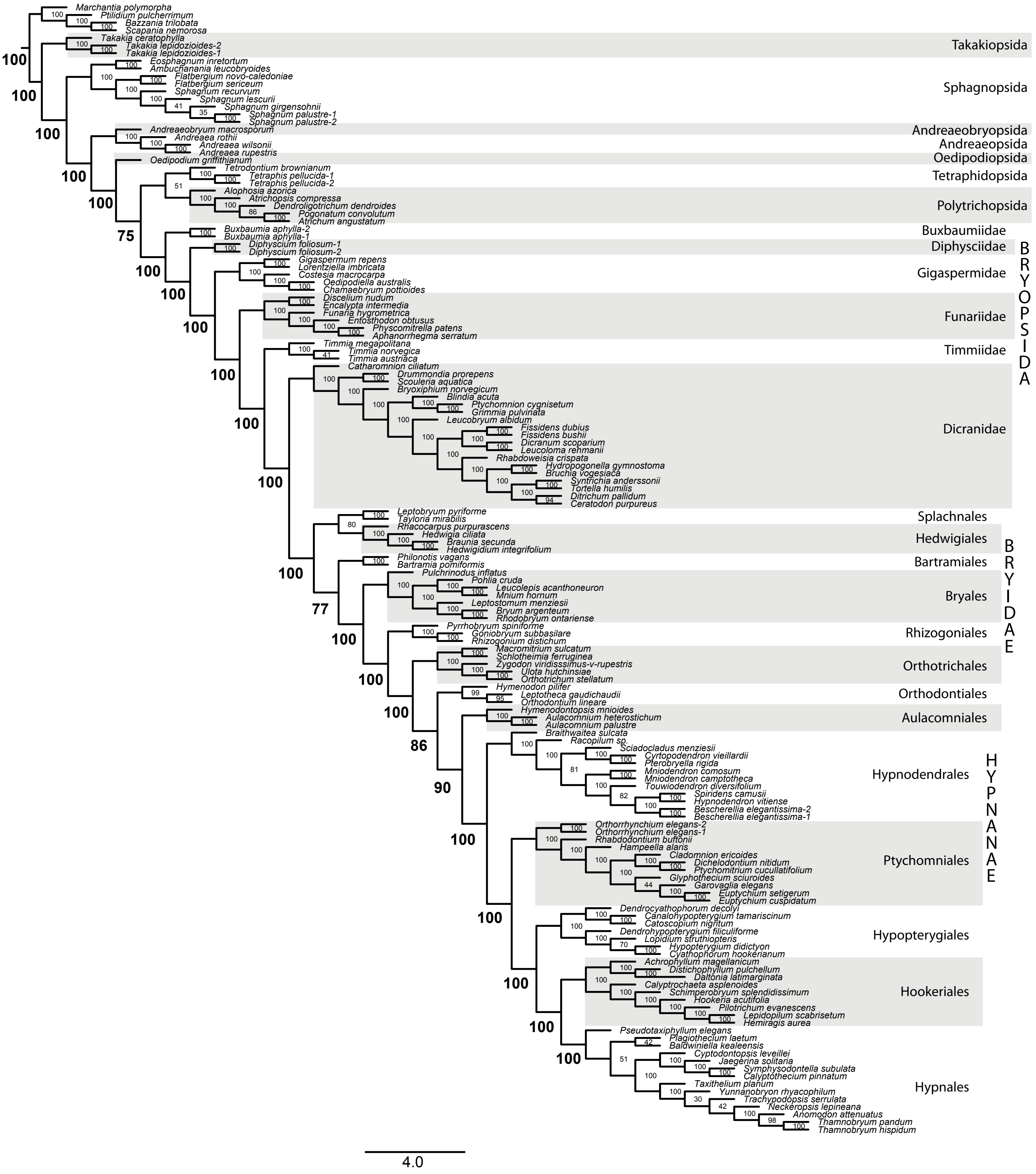

Supplementary Figure 23. Maximum quartet tree from ASTRAL, DNA trees, MLBS Support: The tree is generated from 106 nucleoide RAXML gene trees. Prior to running ASTRAL, gene trees were collapsed to remove bipartitions not supported by at least 33% RAXML bootstrap support. Support values indicate mul locus bootstrap (MLBS), in which the maximum quartet tree was calculated 100   mes using a randomly selected RAXML bootstrap tree instead of maximum likelihood trees for each gene. Branch lengths in coalescent units (2*N generations) and are directly proportional to the amount of discordance

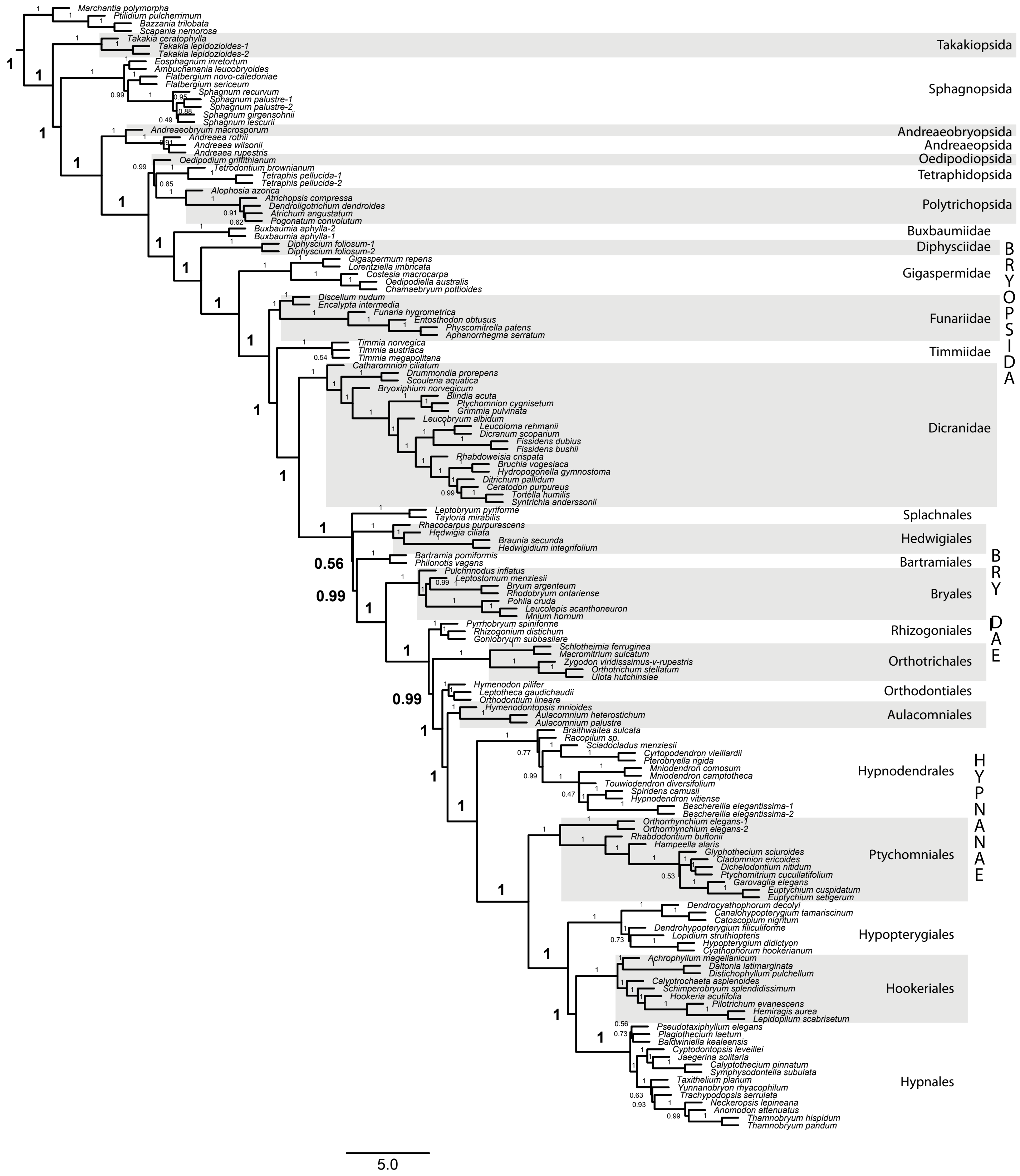

Supplementary Figure 24. Maximum quartet tree from ASTRAL, AA trees, LPP Support: The tree is generated from 106 amino acid RAXML gene trees. Prior to running ASTRAL, gene trees were collapsed to remove bipartitions not supported by at least 33% RAXML bootstrap support. Support values indicate the local posterior probability (LPP), which assesses the support for the quartet tree versus its three alternatives. Branch lengths in coalescent units (2*N generations) and are directly proportional to the amount of discordance.

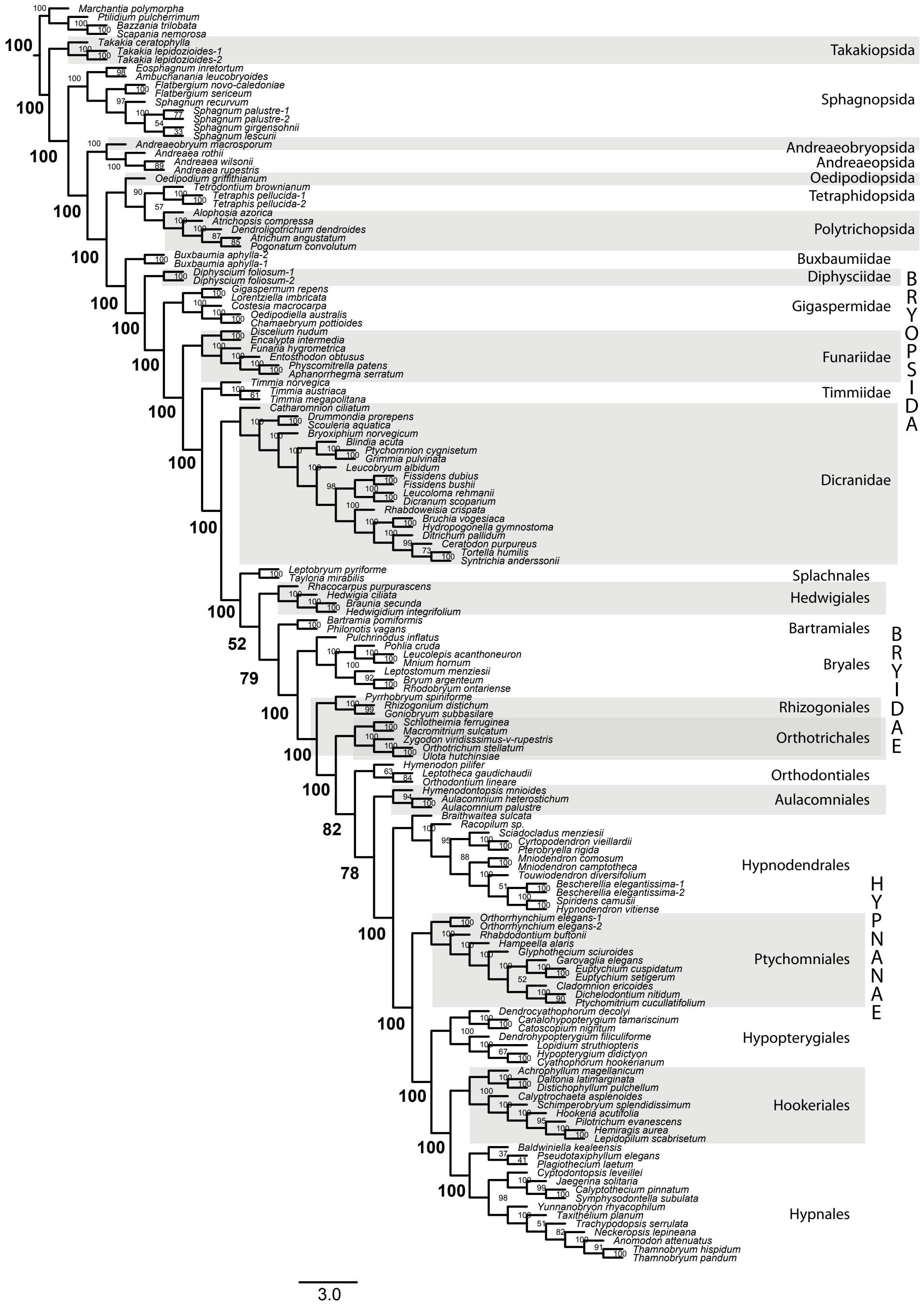

Supplementary Figure 25. Maximum quartet tree from ASTRAL, AA trees, MLBS Support: The tree is generated from 106 amino acid RAXML gene trees. Prior to running ASTRAL, gene trees were collapsed to remove bipartitions not supported by at least 33% RAXML bootstrap support. Support values indicate mul locus bootstrap (MLBS), in which the maximum quartet tree was calculated 100 times using a randomly selected RAXML bootstrap tree instead of maximum likelihood trees for each gene. Branch lengths in coalescent units (2*N generations) and are directly proportional to the amount of discordance.

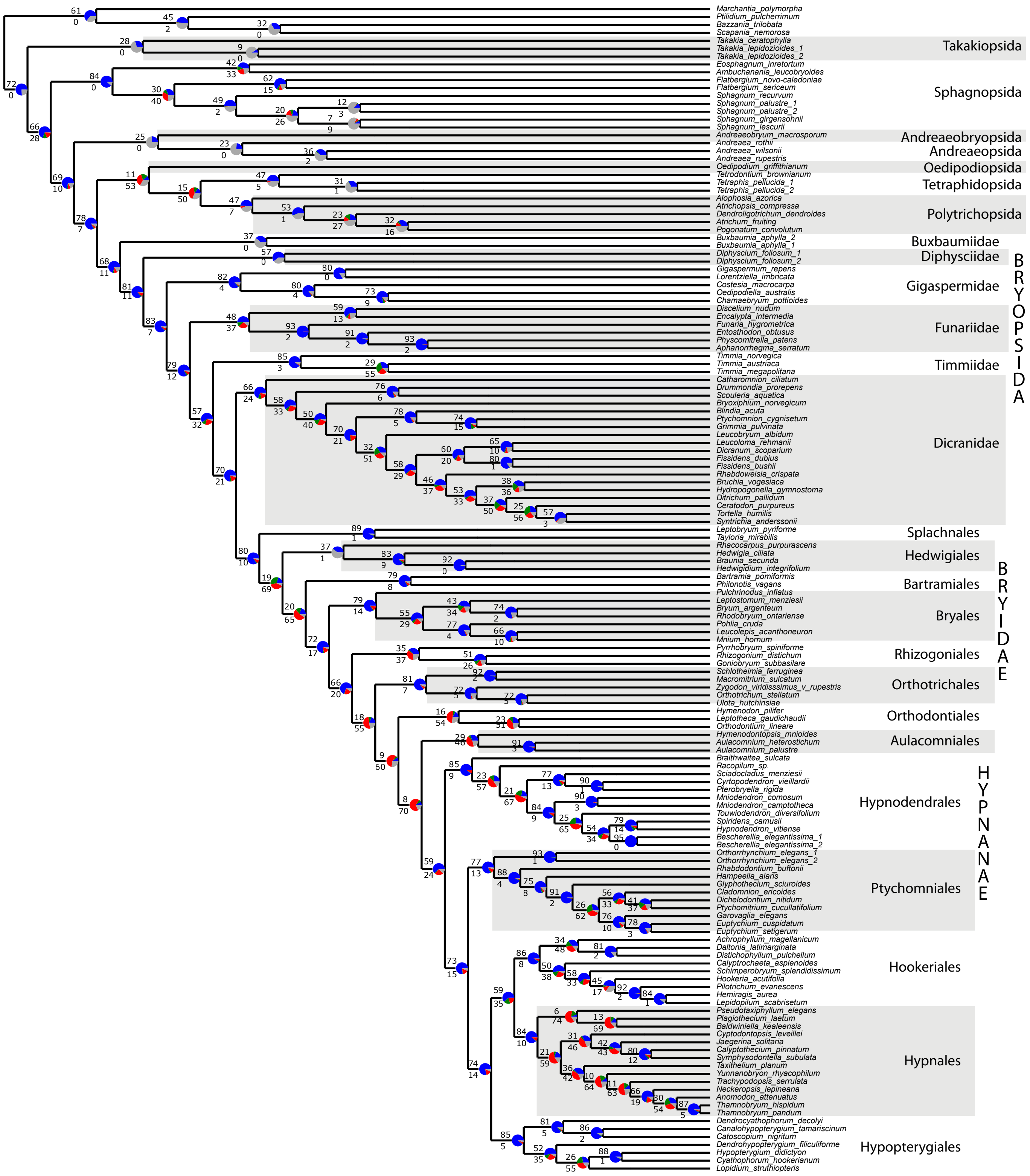

Supplementary Figure 26. Gene tree congruence analysis for 95 nucleotide gene trees compared to the maximum quartet (ASTRAL) tree built from the same data: Bipartitions were calculated using Phyparts (7). Pie charts at each node correspond to the percentage of gene trees that are concordant with the ASTRAL topology (blue), contain the most common alternative bipartition (green), are otherwise discordant from the ASTRAL tree (red) or are uninformative for the given node (gray). The number of gene trees used for this analysis was reduced due to the absence of outgroups in some genes.

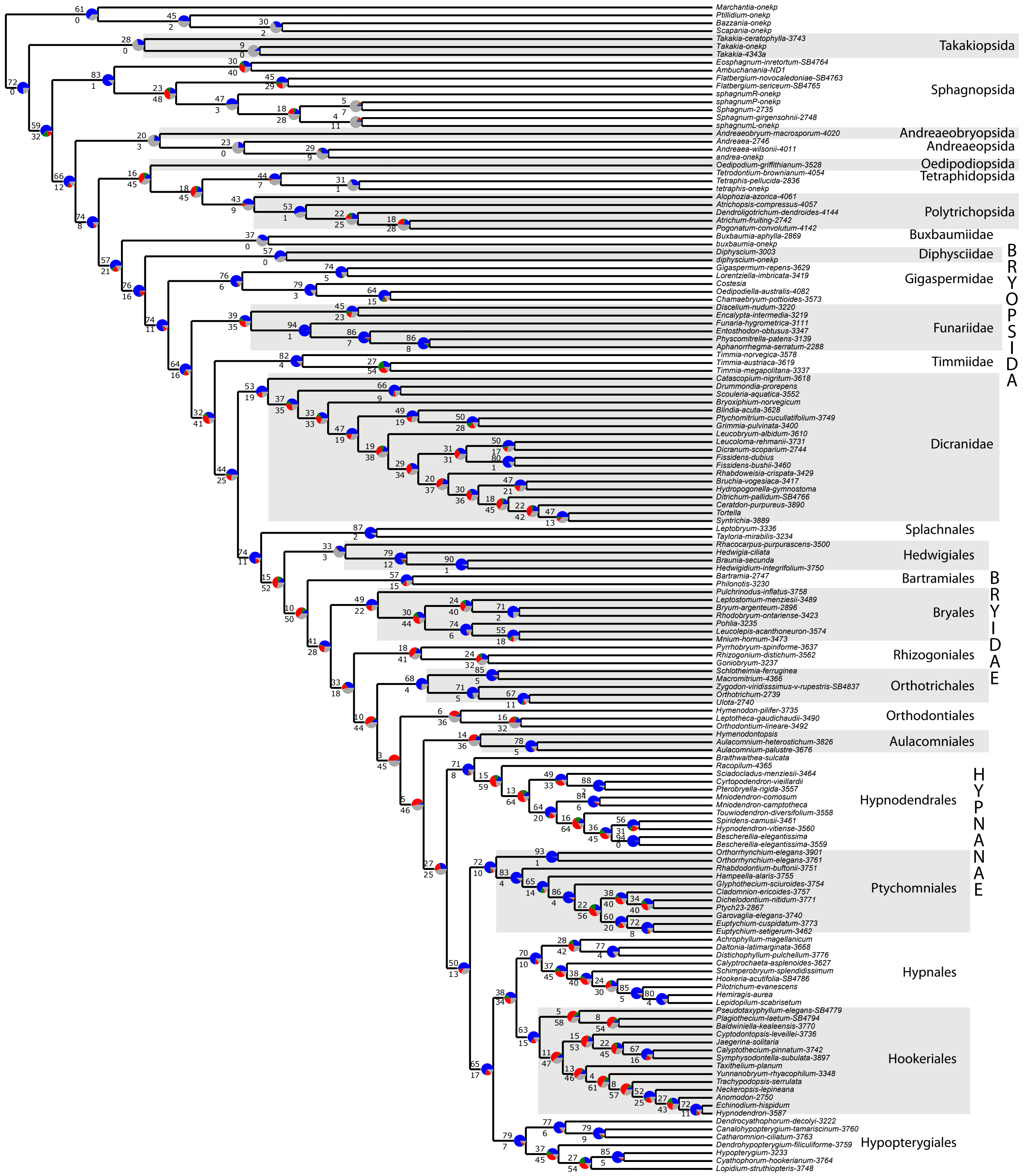

Supplementary Figure 27. Gene tree congruence analysis for 95 amino acid gene trees compared to the maximum quartet (ASTRAL) tree built from the same data: Bipartitions were calculated using Phyparts (7). Pie charts at each node correspond to the percentage of gene trees that are concordant with the ASTRAL topology (blue), contain the most common alternative bipartition (green), are otherwise discordant from the ASTRAL tree (red) or are uninformative for the given node (gray). The number of gene trees used for this analysis was reduced due to the absence of outgroups in some genes.

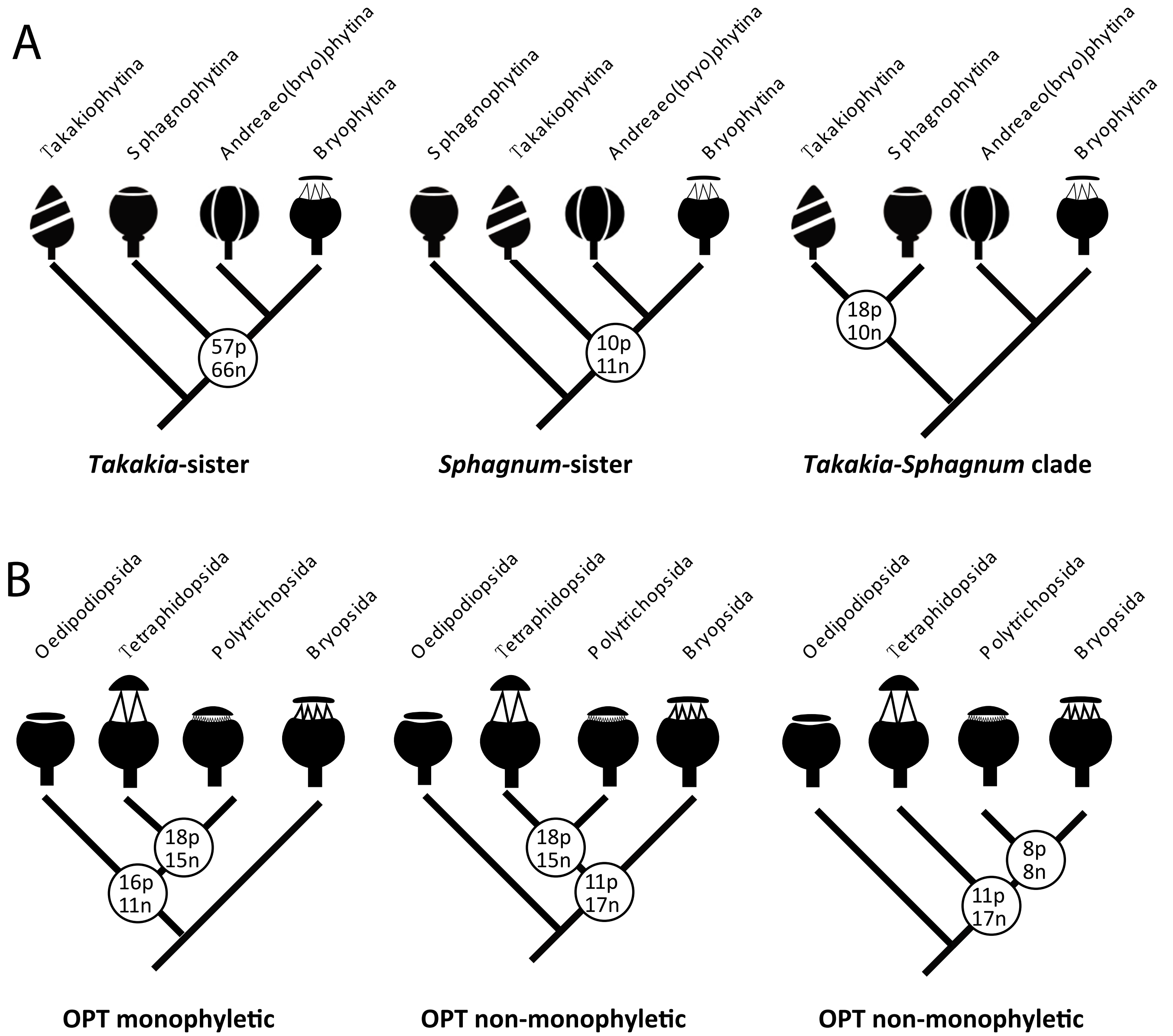

Supplementary Figure 28. Summary of inter- and intra-genomic conflict at two nodes in the Bryophyta phylogeny.

A. The relative position of the four earliest diverging lineages of mosses, which differ primarily in the method sporophyte degeneration for spore dispersal: Takakiopsida (single spiral suture), Sphagnopsida (explosive dehiscence), Andreao(bryo)phytina (four sutures), and Bryophytina (peristome-mediated dispersal). B. The relative position of the four main lineages of early peristomate lineages of mosses differing in the structure of the peristome teeth: Bryopsida (mosses with an arthrodontous peristome), Oedipodiopsida (no peristome), Tetraphidopsida (mosses with a nematodontous peristome of four large teeth) and Polytrichopsida (mosses with typically a nematodontous peristome of many teeth). In each case, the three most likely topologies are shown, with the concordance among single nuclear loci shown at specific nodes (n=DNA gene trees; p=peptide gene trees).

References

1. Goffinet B, Buck WR, Shaw AJ. Morphology, anatomy, and classification of the Bryophyta. In: *Bryophyte Biology, second ed.* (eds Goffinet B, Shaw AJ). Cambridge University Press (2009).

2. Stech M, *et al.* Explaining the 'anomalous' distribution of *Echinodium* (Bryopsida: Echinodiaceae): independent evolution in Macaronesia and Australasia. *Organisms Diversity & Evolution* **8**, 282–292 (2008).

3. Matasci N, *et al.* Data access for the 1,000 Plants (1KP) project. *GigaScience* **3**, 17 (2014).

4. Devos N, Szövényi P, Weston DJ, Rothfels CJ, Johnson MG, Shaw AJ. Analyses of transcriptome sequences reveal multiple ancient large-scale duplication events in the ancestor of Sphagnopsida (Bryophyta). *New Phytologist* **211**, 300–318 (2016).

5. Rensing SA, *et al.* The Physcomitrella genome reveals evolutionary insights into the conquest of land by plants. *Science* **319**, 64–69 (2008).

6. Drouin G, Daoud H, Xia J. Relative rates of synonymous substitutions in the mitochondrial, chloroplast and nuclear genomes of seed plants. *Molecular Phylogenetics and Evolution* **49**, 827–831 (2008).

7. Smith SA, Moore MJ, Brown JW, Yang Y. Analysis of phylogenomic datasets reveals conflict, concordance, and gene duplications with examples from animals and plants. *BMC Evolutionary Biology* **15**, 150 (2015).
